# Intratumoral Biosynthesis of Gold Nanoclusters by Pancreatic Cancer to Overcome Delivery Barriers to Radiosensitization

**DOI:** 10.1021/acsnano.3c04260

**Published:** 2024-01-11

**Authors:** Aaron S. Schwartz-Duval, Yuri Mackeyev, Iqbal Mahmud, Philip L. Lorenzi, Mihai Gagea, Sunil Krishnan, Konstantin V. Sokolov

**Affiliations:** † Department of Imaging Physics, The University of Texas MD Anderson Cancer Center, 1515 Holcombe Boulevard, Houston, Texas 77030, United States; ‡ Vivian L. Smith Department of Neurosurgery, University of Texas Health Science Center, Houston, Texas 77030, United States; § Department of Bioinformatics and Computational Biology, The University of Texas MD Anderson Cancer Center, 1515 Holcombe Boulevard, Houston, Texas 77030, United States; ⊥ Department of Veterinary Medicine & Surgery, The University of Texas MD Anderson Cancer Center, 1515 Holcombe Boulevard, Houston, Texas 77030, United States

**Keywords:** biomineralization, gold nanoparticles, *in situ* therapies, radiosensitization, pancreatic cancer

## Abstract

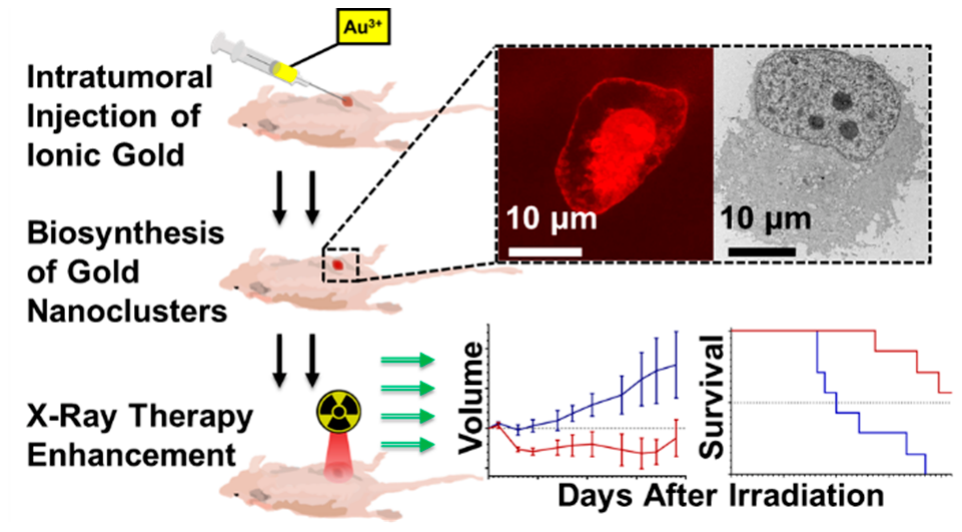

Nanoparticle delivery to solid tumors is a prime challenge in nanomedicine. Here, we approach this challenge through the lens of biogeochemistry, the field that studies the flow of chemical elements within ecosystems as manipulated by living cellular organisms and their environments. We leverage biogeochemistry concepts related to gold cycling against pancreatic cancer, considering mammalian organisms as drivers for gold nanoparticle biosynthesis. Sequestration of gold nanoparticles within tumors has been demonstrated as an effective strategy to enhance radiotherapy; however, the desmoplasia of pancreatic cancer impedes nanoparticle delivery. Our strategy overcomes this barrier by applying an atomic-scale agent, ionic gold, for intratumoral gold nanoparticle biosynthesis. Our comprehensive studies showed the cancer-specific synthesis of gold nanoparticles from externally delivered gold ions *in vitro* and in a murine pancreatic cancer model *in vivo*; a substantial colocalization of gold nanoparticles (GNPs) with cancer cell nuclei *in vitro* and *in vivo*; a strong radiosensitization effect by the intracellularly synthesized GNPs; a uniform distribution of *in situ* synthesized GNPs throughout the tumor volume; a nearly 40-day total suppression of tumor growth in animal models of pancreatic cancer treated with a combination of gold ions and radiation that was also associated with a significantly higher median survival versus radiation alone (235 vs 102 days, respectively).

Localized therapies are a critical component of cancer treatment, and there is a renewed interest in innovative ways of intensifying radiotherapy for cancer treatment. The increased toxicity and lack of survival benefit from the elective irradiation of locoregional nodal basins has prompted a shift toward dose-escalation strategies that focus on just the primary tumor.^1^ The initially reported role of dose escalation^2^ was corroborated by a recent multicenter study showing that a 3-week course of dose-escalated hypofractionated radiotherapy significantly improved local control and overall survival.^3^ However, the utility of radiotherapy is limited by the resistance of some cancer cells to radiation. Thus, there is a critical need to develop methods that increase the radiation dose delivered to cancer cells. An emerging approach to enhancing the radiation dose delivered to tumors is to use high atomic number (high-Z) materials such as hafnium oxide^4^ or gold nanoparticles (GNPs)^5−13^ to transiently increase the radiation-interaction probability of the target tissues. This effect is attributed to an increase in photoelectric absorption interactions due to the high Z of gold followed by the greater physical damage to tumor and endothelial cells caused by secondary (photo and Auger) electrons from nanoparticles.^5−13^

Optimum enhancement of RT by GNPs or other high Z nanoparticles (NPs) requires: (1) efficient delivery to the tumor, (2) homogeneous uptake by the cancerous cells, and (3) intranuclear localization. The strategies for delivery of NPs to solid tumors were recently summarized in a comprehensive review by Izci et al.^14^ The inefficiency of delivery of NPs to solid tumors was highlighted by a meta-analysis, suggesting that only ∼0.7% of the total intravenously injected dose accumulates in tumors in preclinical tumor models.^15^ A follow up study by the same group showed that the delivery efficiency in murine cancer models could be increased to 12% if the administered dose exceeds 1 trillion nanoparticles that was attributed to saturation of the ability of Kupffer cells to uptake NPs;^16^ however, the clinical translatability of this concept still needs to be evaluated. Most of nanoparticle delivery strategies to solid tumors rely on the enhanced permeability and retention (EPR) effect.^17^ Maximization of the EPR effect for delivery requires tailoring the nanoparticle size and surface coating to optimize the relationship between circulation and clearance, as well as preventing recognition and clearance by immune cells.^14,18−20^ However, translation of EPR-reliant approaches could be severely limited by both the intra- and interpatient heterogeneity of this effect in the tumors of human patients.^21^ Because of the heterogeneity of the EPR effect, many studies have explored strategies to modify the tumor vascularization including permeabilization, normalization, disruption, or promotion of vascularization to enhance treatment efficiency.^22^ However, a response to these *in situ* vascular modification strategies can be variable between patients, might be associated with additional side effects, and would require additional spatial and temporal control.^22^ To enhance the retention component of the EPR effect, many researchers have applied active targeting strategies reliant on ligand binding.^23−25^ However, a recent study indicated that only ∼2% of targeted NPs interact with cancer cells at the tumor site.^26^ This observation could be associated with nanoparticle uptake by other cells in the tumor microenvironment such as tumor associated macrophages and fibroblasts.^27,28^ Further, the tumor expression profiles are often highly heterogeneous. To mitigate this problem, some studies have explored combinations of multiple targeting ligands on the same nanoparticle;^29^ however, this strategy complicates the NP’s synthesis as the number of targeting antibodies needs to be controlled in order to maintain targeting efficiency.^30^

Recent biomimetic strategies for nanoparticle delivery use extracted cell membrane or membrane derived extracellular vesicles (EVs).^31−44^ Membrane-coated NPs derived from blood cells were shown to significantly improve blood circulation time and even the ability to home to tumors via inflammation associated pathways.^31−35,43^ NPs coated with cancer-derived cell membranes “inherit” the cancer cells’ ability to evade immune detection in combination with homotypic adhesion properties for tumor targeting.^36−38^ However, cell membrane coating strategies are complicated by difficulties in acquiring and storing the source material in high quantities.^39−41^ Similar to membrane coated NPs, EVs have shown considerable promise in immune escape and tumor targeting properties.^45−47^ However, the translation of EVs to clinical applications is currently limited by isolation procedures, which have low purity, low yield, and low loading capacity.^47^

Moreover, in most cases, NPs are confined to cellular endosomal compartments following intracellular uptake that limits their radiosensitization potential.^48,49^ An additional challenge facing nanoparticle delivery, even for advanced targeted approaches, is that many solid tumors are characterized with an exuberant interstitial matrix of glycosaminoglycans, collagen, and proteoglycans (i.e., desmoplasia) that serves as a physiological barrier to the delivery of even very small nanoparticles.^14,50^ Because the stroma also confines cancer cells to the tumor, depleting the stroma, and thus risking metastasis, may not be an effective strategy for improved nanoparticle delivery and radiation dose escalation.^51,52^

To address these delivery challenges, we explored a radiosensitization strategy where ionic gold (Au^3+^) is used as a precursor for the *in situ* biomineralization of GNPs and gold nanoclusters (GNCs) within the tumor. Changing the current paradigm from the delivery of premade GNPs, which are 5–200 nm, to the delivery of Au^3+^, which are approximately 0.3 nm, is associated with an ∼4.6 × 10^3^ to 3 × 10^8^ reduction in volume of a gold therapeutic agent that is much more likely to uniformly diffuse throughout a desmoplastic tumor microenvironment. This strategy is founded on recent reports of mammalian cells’ biomineralization of GNPs.^53−66^ These studies showed the colocalization of intracellularly formed nanoparticles within the cells’ nuclei^53−55,65^ and indicated that GNP biomineralization from the application of chloroauric salts occurs more readily in cancer cells than in normal cells.^56−59,61^ They also provided evidence of intratumoral gold biomineralization in xenograft mouse models.^56,58,65,67^ In addition, injectable gold-salt solutions have been used safely for more than 80 years in the treatment of rheumatoid arthritis.^68^ Furthermore, the prolonged use of injectable gold-salt drugs was found to lead to a mild side effect (i.e., chysiasis) due to in-patient GNP formation,^69−71^ thus providing additional proof of the feasibility of *in situ* gold biomineralization. However, *in situ* gold biomineralization has not been considered for applications in radiotherapy or thoroughly evaluated for other clinically translatable applications.

In the present study, we evaluated a gold biomineralization-based radiosensitization strategy in a model of pancreatic cancer, an aggressive malignancy whose yearly incidence nearly equals its mortality rate and the classic example of a recalcitrant, difficult-to-treat tumor.^72^ In the present study, we characterized cellular biosynthesis of GNCs, finding preferential Au^3+^ uptake and particle formation, with innate nuclear localization, by cancerous compared against normal cells. We then optimized the Au^3+^ treatment conditions to maximize particle formation with minimal impact to cell viability. The optimized treatment was used for mechanistic studies of ion internalization and cancer cell radiosensitization including DNA repair disruption, metabolic dysregulation, and lipid breakdown, which we further interrogated using combined lipidomic and metabolomic strategies. Finally, we quantified biodistribution, toxicity, and radiosensitization *in vivo* using a xenograft mouse model of pancreatic cancer, finding strong tumor colocalization of *in situ* formed GNPs, minimal treatment-related toxicity, and a strong radiosensitization effect.

## Results and Discussion

### Intracellular Gold Reduction by Pancreatic Cells

**Figure 1:**
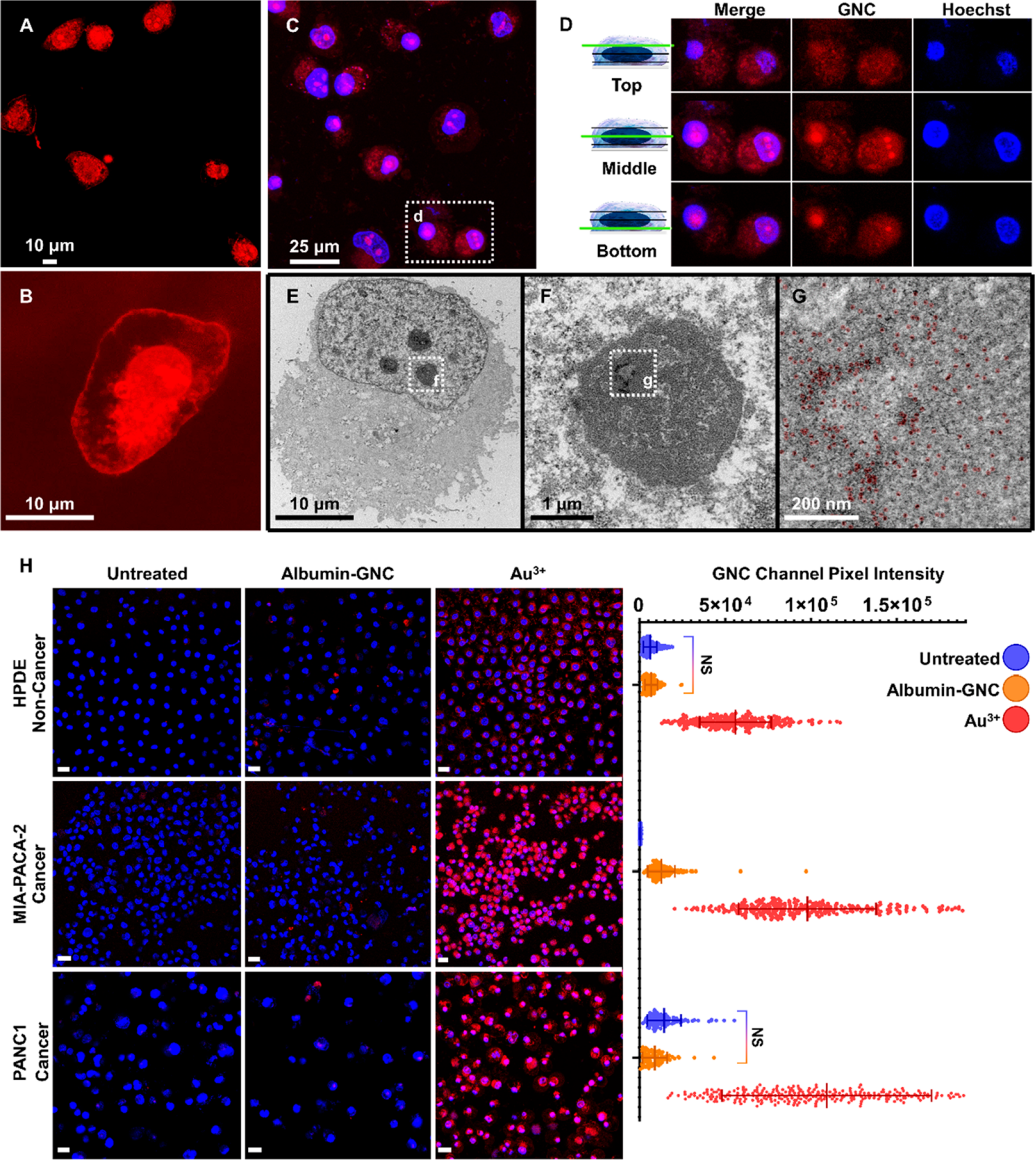
*In situ* GNP biomineralization in pancreatic cancer cells. (A, B) Confocal fluorescence images of intracellularly formed GNCs (_ex_561/_em_610 nm) in live PANC1 cells after 24 h of treatment with 1.0 mM chloroauric acid without additional staining. (C, D) Maximum projection image (17 slices; C) and cross-sectional confocal images (D) of the top, middle, and bottom optical slices of live PANC1 cells showing intracellularly formed fluorescent GNCs (red) overlaid with Hoechst 33342 nuclear staining (blue) after 24 h of treatment with 1.0 mM chloroauric acid. (E–G) TEM images of PANC1 cells after 24 h of treatment with 1.0 mM chloroauric acid (E); magnified images of the nucleoli showing GNCs highlighted in red (F, G). (H) Confocal fluorescence images of the GNC signal (red) overlaid with Hoechst 33342 nuclear staining (blue) in live HPDE, Mia-PaCa-2, and PANC1 cells after 24 h of treatment with 1.0 mM gold as either prefabricated albumin-coated GNCs (Albumin-GNC) or chloroauric acid (Au^3+^); untreated cells were the negative control. Scale bars are 25 μm. The quantification of relative GNC fluorescence is shown on the right; all differences were significant (*P* < 0.0001) except those marked “NS” (not significant; *P* > 0.5; ordinary 1-way ANOVA for multiple comparisons).

For the initial investigation of pancreatic cancer cells’ biomineralization of GNPs, we used common treatment parameters reported previously for other mammalian cells,^73^ i.e., 1.0 mM chloroauric acid (as the source of Au^3+^ gold ions) in full cell media for 24 h. Live-cell confocal microscopy without staining (to eliminate the possibility of stain-induced Au^3+^ reduction) revealed a strong fluorescence signal characteristic of GNC formation^56,58,59,61^ in the Au^3+^ treated cells (Figure 1A,B) that was absent in the untreated cells (Figure S1). The fluorescence was detected in structures whose appearances were consistent with cellular and nuclear membranes, intracellular vesicles, and nuclei (Figure 1B). Emission spectra obtained with 561 nm laser excitation from different subcellular locations (i.e., cytoplasmic membrane, cytosol and nuclei) revealed peaks around 600–610 nm (Figure S1b,c) with the fluorescence intensity from GNCs in the nucleoli ∼125-fold greater than the cytosolic or membrane fluorescence (Figure S1b,c). Subsequent cross-sectional confocal imaging with Hoechst 33342 nuclear staining and transmission electron microscopy (TEM) confirmed the intranuclear localization of GNCs in the treated cells (Figure 1C–G). The intranuclear fluorescence from GNCs exhibited a pattern with bright loci that was consistent with nucleoli. TEM images showed consistently higher mean electron density values for the nucleoli of treated PANC1 cells, with identifiable GNCs with a mean size of 3.1 ± 1.8 nm (*n* = 2196 particles) (Figure S2a–f).

**Figure 2:**
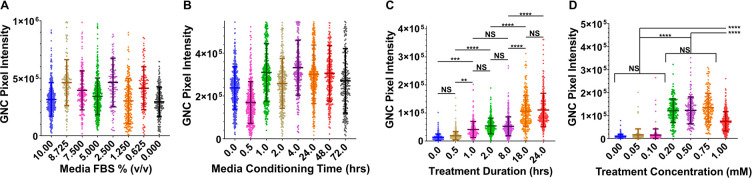
Optimization of *in situ* GNP biomineralization. (A–D) Quantification of total intranuclear GNC channel pixel intensity per cell, measured from confocal fluorescence images (*n* = 3 images) of live PANC1 cells after treatment with chloroauric acid in cell media with various FBS concentrations (% v/v; A), media conditioning times (B), chloroauric acid treatment durations (C), or chloroauric acid treatment concentrations (D). Standard treatment conditions were 10% (v/v) FBS; 24 h of media conditioning by cells before treatment with chloroauric acid; 24 h of treatment with chloroauric acid; treatment with 1.0 mM chloroauric acid. Representative confocal images of the cells used in these analyses are provided in Figure S3. Error bars are standard deviations. ^NS^*P* > 0.05, *P < 0.05, **P < 0.01, ***P < 0.001, and ****P < 0.0001; ordinary 1-way ANOVA for multiple comparisons.

Next, we compared the cellular biomineralization of GNCs with uptake of prefabricated albumin-coated GNCs (Figure S2g,h) in two pancreatic cancer cell lines (PANC1 and Mia-PaCa-2)^74^ and in noncancerous human pancreatic duct epithelial (HPDE) cells. Confocal fluorescent imaging confirmed that both cancer cell lines had GNC biomineralization, which occurred with approximately 2-fold greater efficiency than in the noncancer cells (*P* < 0.0001 for both comparisons) (Figure 1H). Compared with prefabricated albumin-coated GNCs, Au^3+^ treatments resulted in 12- and 7-fold greater fluorescence in PANC1 and Mia-PaCa-2 cells, respectively (Figure 1H). These findings indicate that the albumin in the cell culture media does not enable extracellular biomineralization with subsequent uptake of extracellularly formed GNCs in Au^3+^-treated cells. Indeed, if the extracellular albumins were responsible for reducing gold ions, then the cells treated with prefabricated albumin-coated GNCs would have had a fluorescence signal comparable to that from the cells treated with gold ions. Further confirming this conclusion, we found that supplementing the incubating cell media with various amounts of fetal bovine serum (FBS) before treatment with Au^3+^ was not associated with any consistent trend in GNC fluorescence from biomineralization (Figures 2A and S3a).

### Optimization of Treatment Conditions for Intracellular GNC Biomineralization

We investigated how cell secretions, the Au^3+^ treatment duration, and the Au^3+^ concentration influence the fluorescence signal from GNCs formed inside PANC1 cells (Figure 2B–D, Supporting Information, and Figure S3b–d) and then interrogated how those variables influence cellular viability (Figure S4f–i). Media preconditioning with PANC1 cells up to 72 h did not reveal any identifiable trends in intranuclear fluorescence of GNCs (Figure 2B). The fluorescence signal steadily increased after 1 h of incubation with Au^3+^ ions achieving the maximum value of ∼18 h (Figure 2C). Concentrations of 0.20–0.75 mM Au^3+^resulted in the greatest fluorescence intensity (Figure 2D) while Au^3+^ treatments with ≤0.20 mM did not significantly impact PANC1 cell viability (Figure S4f–i). Based on these optimization studies, the following conditions for Au^3+^ treatment in cell culture were used for subsequent studies unless otherwise stated: 24 h incubation of cells in media with 10% (v/v) FBS followed by 0.20 mM Au^3+^ treatment for 24 h.

### Interrogation of Gold Ion Uptake

The specific mechanisms of gold ion entry in mammalian cells are not fully understood. However, prior studies indicate that Au^3+^ uptake occurs through mechanisms distinct from classical nanoparticle pathways and that pathways for Au^3+^ uptake are more efficient than the classical pathways, which are common for prefabricated nanoparticles.^60,65^ Schwartz-Duval et al.^65^ used a combination of molecular inhibitors and genomic studies to demonstrate that the classical nanoparticle uptake pathways (i.e., energy-dependent-, dynamin-dependent-, lipid raft-, clathrin-dependent-, and clathrin-independent-based uptakes) were not involved in the uptake of gold ions. Additionally, these studies showed no significant difference in gold uptake between cells with inhibited endocytosis pathways and controls that suggests a negligible uptake of extracellularly formed nanoparticles, which commonly occurs through various endocytosis mechanisms.^65^ The latter result was supported by Drescher et al.^60^ who directly compared the uptake of gold ions with prefabricated particles and observed a much greater cellular uptake of gold ions. Our data in Figure 1H are in a good agreement with these reports as we observed a negligible uptake of prefabricated GNCs compared to *in situ* biomineralization of GNCs by pancreatic cells. We also determined that neither serum albumins nor secreted biomolecules are majorly involved in shuttling Au^3+^ inside cells as there were no clear dependence of GNC intracellular formation on either concentration of FBS or media conditioning time, respectively (Figure 2A,B).

Therefore, we decided to explore the feasibility of involvement of cellular ion channels in the uptake of gold ions. To test this hypothesis, we coapplied titrated dosages of physiologically relevant cations with atomic radii comparable to Au^3+^ (i.e., Mg^2+^, Ca^2+^, Mn^2+^, Fe^3+^, and Fe^2+^) at high and medium Au^3+^ concentrations (i.e., 1.0 and 0.5 mM, respectively). GNC fluorescence was used to assay *in situ* gold biomineralization. Among the selected cations, magnesium has the greatest intracellular abundance (10–30 mM)^75^ while the other cations (i.e., Ca^2+^, Mn^2+^, Fe^3+^, and Fe^2+^) are present at lower concentrations. The physiological cation concentrations were used to set the titration doses.

**Figure 3:**
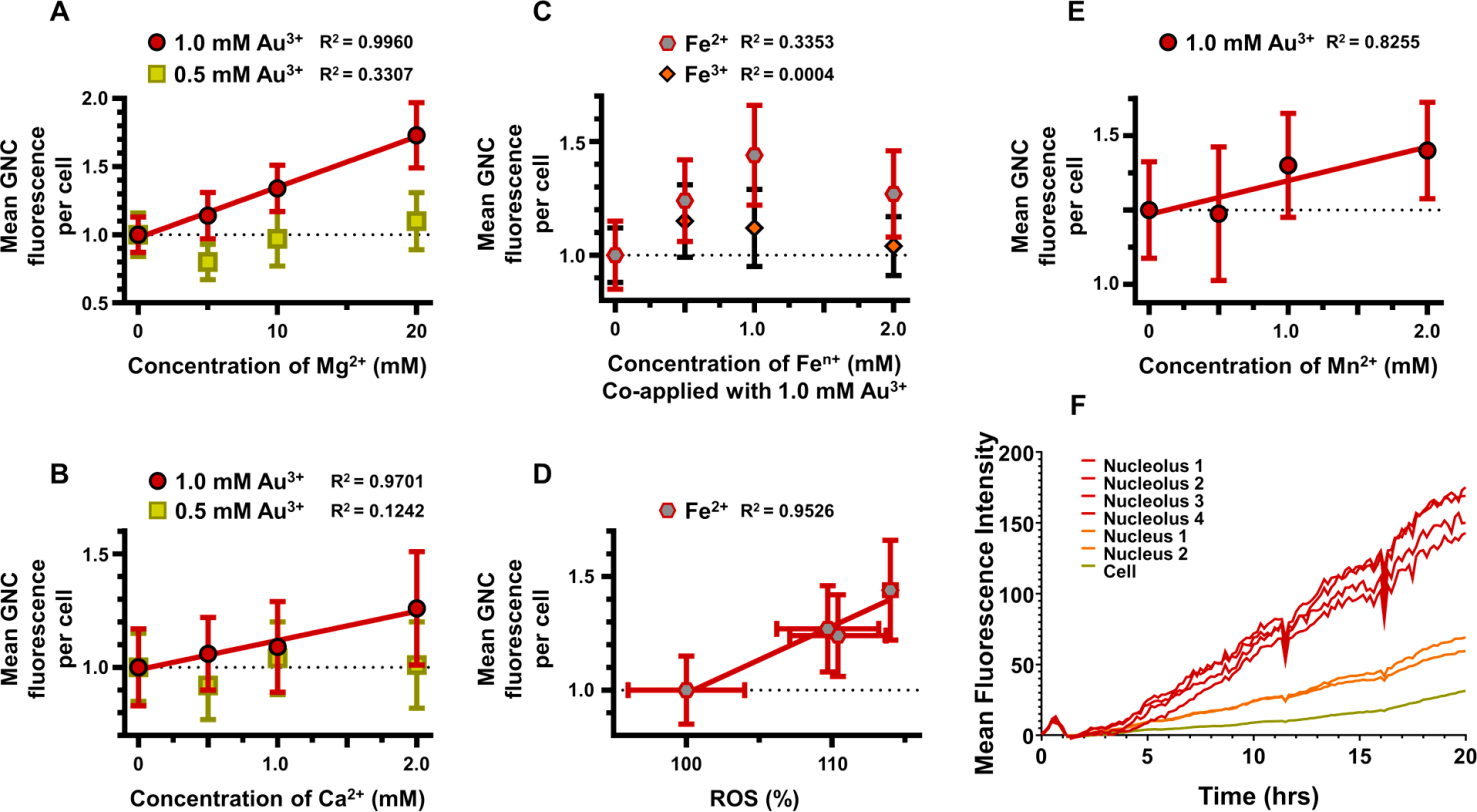
Interrogation of gold ion uptake for *in situ* GNC biomineralization. (A–E) Plots showing relationships between mean GNC fluorescence from Au^3+^ biomineralization and coapplications of physiological cations Mg^2+^ (A), Ca^2+^ (B), Fe^2+^ and Fe^3+^ (C), ROS formation from Fe^2+^ treatment (D) and Mn^2+^ (E) over a 24 h period at varied physiologically relevant cation concentrations in PANC1 cells. (F) Mean fluorescence intensity measurements collected once every 10 min for 20 h for specific regions of interest, such as the cellular nucleolus, nucleus, and whole cell, during treatment of PANC1 cells with 0.20 mM chloroauric acid in full cell media under normal incubation conditions. Fluorescence data points and error bars (standard deviations, *n* = 3) are from confocal microscopy images.

We observed that cotreatments with Mg^2+^ and Ca^2+^ ions (Figure 3A,B, respectively) resulted in a linear increase in GNC fluorescence at 1.0 mM Au^3+^ while there was no correlation at 0.5 mM Au^3+^. A prior study by Zhao et al.^58^ showed that biomineralization of GNCs from Au^3+^ could be enhanced by increasing intracellular ROS formation that was induced by the addition of Fe^2+^ ions. Therefore, we evaluated ROS formation and metabolic cell viability at Mg^2+^ and Ca^2+^ concentrations used in our study and found no substantial changes in either parameter that excludes the possibility of ROS involvement in the observed increase in GNC biomineralization (Figure S5a–d). In addition, we showed that cell cotreatment with Fe^2+^ ions results in an increase in GNC fluorescence (Figure 3C) and Fe^2+^ treatment induces ROS production (Figures 3D and S5e,f), which is in agreement with the study by Zhao et al.^58^ Cell cotreatment with Fe^3+^ showed no correlation with GNC fluorescence (Figure 3C), and there was no substantial effect of Fe^3+^ treatment on either cell viability or ROS production (Figure S5g,h). We also found a linear correlation between Mn^2+^ supplement and GNC fluorescence (Figure 3E); however, this correlation may be influenced by the impact Mn^2+^ had on cellular viability rather than ROS induction (Figure S5i,j).

Data in Figure 2D indicate that GNC fluorescence decreases when the concentration of Au^3+^ is increased to 1.0 mM. Therefore, the positive correlation between the concentration of Mg^2+^ and Ca^2+^ ions and GNC fluorescence at 1.0 mM Au^3+^ might indicate a competition for cellular entry pathways that effectively decreases gold ion uptake resulting in an increase of GNC fluorescence. The lack of correlation between cotreating cations and the GNC fluorescence at 0.5 mM could be due to the plateau in GNC fluorescence between 0.2 and 0.75 mM Au^3+^, suggesting that a decrease in effective Au^3+^ concentration below 0.5 mM, but above 0.2 mM, would not be associated with a GNC fluorescence change (Figure 2D). Therefore, our cation cotreatment study suggests that Mg^2+^ and Ca^2+^ ions and their channels as interesting candidates for future exploration of Au^3+^ cell uptake and *in situ* gold biomineralization in general.

The total gold content, measured by ICP-MS under optimized conditions, showed Mia-PaCa-2, PANC1, and HPDE cells had uptakes of the total gold dose of 18.3 ± 2.1%, 19.0 ± 3.6%, and 13.7 ± 1.2%, respectively (Figure S6a), linearly correlated (*r*^2^ = 0.57, *P* = 0.0185) with the intranuclear GNC fluorescence shown in Figure 1H (Figure S6b). Interestingly, the difference in intracellular synthesis of GNCs between the cancerous and noncancerous cells as assessed by fluorescence is significantly more pronounced than the difference between the total gold uptake from ICP-MS.

Next, we evaluated the locoregional time dependence of *in situ* biomineralization of GNCs and the potential intracellular trafficking of the formed GNCs into nuclei using longitudinal live cell imaging, as well as characterized the labeling efficiency at the 24 h time point under optimized treatment conditions. For longitudinal live cell imaging, we transfected PANC1 cells with a Bacman Cell light nuclear GFP kit for initial localization of nuclei in confocal fluorescent live cell imaging. Then, we carried out the real-time visualization of the cellular biomineralization process (Supplementary Video 1). GNC fluorescence emerged simultaneously in the nucleus, nucleolus, nuclear membrane, cytosol, and cell membrane of all cells uniformly without any detectable fluorescence in the extracellular space. The overall fluorescence pattern did not change over time and had the same appearance as in the final images shown in Figure 1A–D. The fluorescence intensity steadily increased at different rates throughout the cell, with the highest and lowest rates observed in the nucleoli and cytosol, respectively (Figure 2F). These findings indicate that, within the time period explored, gold ions are able to permeate the cell unencumbered and without any transportation or trafficking of intracellularly formed GNCs. They also suggest that fluorescent GNC biomineralization occurs primarily through interactions within the cell and not through interactions with extracellular cell secretions. However, it is conceivable that some nonfluorescent GNPs could have formed within the extracellular space and were not detected by fluorescence imaging.

Flow cytometry showed ∼68% PANC1 cells with a positive signal from GNCs following a 24 h treatment with 0.20 mM Au^3+^ in full cell media (Figure S6c). This result does not appear to fully correlate with our confocal fluorescence imaging where we observed the near uniform biosynthesis of GNCs by confocal imaging shown in Figures 1 and S3 and Supplementary Video 1. This discrepancy could be related to heterogeneity in biosynthesis of GNCs inside cells with ∼7-fold greater fluorescence from nucleoli relative to the entire cell (Figures 3F and S1). Therefore, a strong localized increase in nucleoli fluorescence is very prominent in confocal fluorescence microscopy, but in some cells, it might not result in an appreciable increase in the overall cell fluorescence that is detected by flow cytometry.

### Radiosensitization of Pancreatic Cancer Cells by GNCs Synthesized *in Situ*

**Figure 4:**
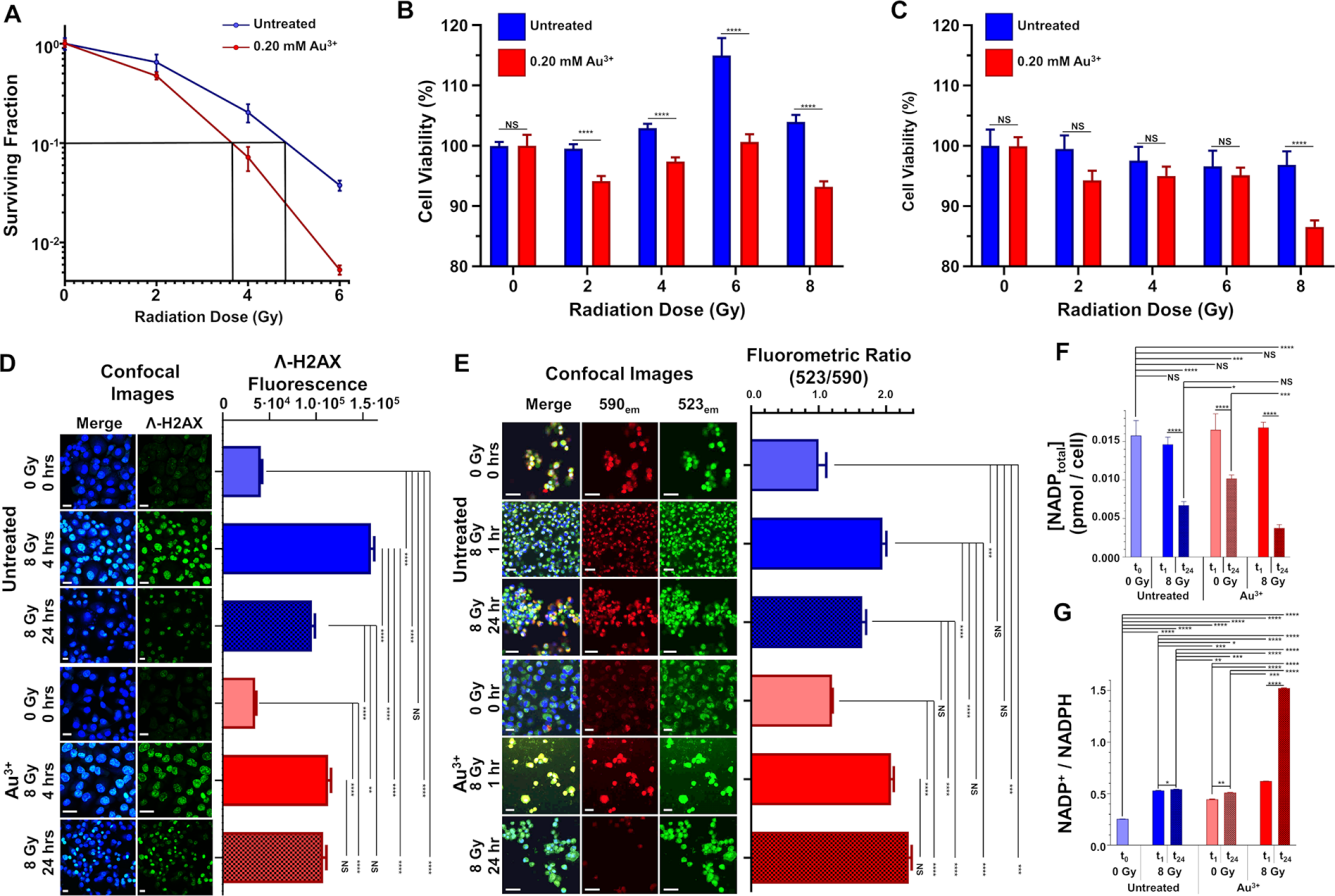
Radiosensitizing effects of GNCs biomineralized *in situ*. (A) A clonogenic assay of PANC1 cells treated with or without 0.20 mM Au^3+^ and exposed to 0–6 Gy of X-ray radiation. The black lines intersecting the *x*-axis represent the radiation dose necessary to reduce the surviving fraction to 10%. (B, C) MTS of PANC1 cells treated with or without 0.20 mM Au^3+^ and exposed to 0–8 Gy of X-ray radiation at 24 h after irradiation (B) and 96 h after irradiation (C). (D) Confocal fluorescence images of double-stranded DNA breaks detected with fluorescent γ-H2AX antibody staining (green) overlaid with Hoechst 33342 nuclear staining (blue) in fixed PANC1 cells after treatment with Au^3+^ (0.00 or 0.20 mM) and X-ray radiation (0 or 8 Gy) at 0, 4, and 24 h after irradiation. The quantification of γ-H2AX fluorescence is shown on the right. (E) Confocal fluorescence images of relative mitochondrial polarization detected using fluorescent JC-1 staining (590 emission, red; and 523 emission, green) overlaid with Hoechst 33342 nuclear staining (blue) in live PANC1 cells treated with Au^3+^ (0.00 or 0.20 mM) and radiation (0 or 8 Gy) at 0, 1, or 24 h after irradiation. The quantification of the JC-1 fluorometric ratio (590/523) is shown on the right. The scale bars in (D) and (E) are 25 μm. (F, G) Total NADP (F) and NADP^+^/NADPH ratios (G) of PANC1 cells after treatment with Au^3+^ (0.00 or 0.20 mM) and radiation (0 or 8 Gy) at 0, 1, or 24 h after irradiation. Error bars are standard deviations. ^NS^*P* > 0.05, *P < 0.05, **P < 0.01, ***P < 0.001, and ****P < 0.0001; ordinary 1-way ANOVA for multiple comparisons.

We used a standard clonogenic assay to evaluate the radiosensitization efficacy of intracellularly synthesized GNCs in PANC1 cells using the optimized treatment parameters described above (Figure 4A). At radiation doses of 2, 4, and 6 Gy, the mean surviving fractions of PANC1 cells treated with Au^3+^ (47.3%, 7.2%, and 0.5%, respectively) were significantly lower than those of untreated control cells (64.9%, 20.3%, and 3.8%, respectively; *P* < 0.0005), and the dose enhancement factor at a surviving fraction of 10% was 1.317, indicating a strong radiosensitization in Au^3+^-treated cells. In addition, we used an MTS assay to assess the short-term (i.e., 24 and 96 h) effects of radiosensitization on cell viability (Figure 4B,C). At 24 h, we noted that cells treated with 4, 6, or 8 Gy of radiation alone had increased viability, possibly due to radiation-stimulated proliferation,^76−78^ which is not necessarily reflected in a colony-counting clonogenic assay.^76^ However, all Au^3+^-treated cells had significantly decreased viability compared with their radiation only treated counterparts (Figure 4B). At 96 h, all groups except the Au^3+^-treated cells irradiated with 8 Gy had returned to baseline viability (Figure 4C). Together, these findings indicate that 8 Gy of radiation combined with Au^3+^ induces significant short-term radiosensitization-based inhibition of cell viability and proliferation.

To characterize the mechanisms of radiosensitization, we first assessed the formation of double-strand DNA breaks using a γ-H2AX antibody assay.^79^ Four hours after irradiation, Au^3+^-treated cells had 29% fewer DNA breaks than untreated cells (Figure 4D). After 24 h, however, the untreated cells had a significant recovery of DNA breaks (i.e., they had ∼32% fewer breaks at 24 h than at 4 h; *P* < 0.0001) compared with the treated cells, which did not have a recovery of DNA breaks between 4 and 24 h (*P* = 0.72) (Figure 4D). Next, we used a tetraethylbenzimidazolylcarbocyanine iodide (JC-1) assay to assess mitochondrial depolarization, an early stage marker of apoptosis,^80,81^ and found no significant difference between the Au^3+^-treated and untreated cells 1 h after irradiation with 8 Gy. Compared with the nonirradiated cells, the irradiated cells had an approximately 2-fold-greater green/red fluorescence intensity ratio, indicating their increased mitochondrial depolarization (Figure 4E). Twenty-four hours after irradiation, the fluorescence intensity ratio of the untreated cells had decreased to 1.65 ± 0.06, indicating a partial recovery of mitochondrial polarization, whereas that of the Au^3+^-treated cells had increased to 2.34 ± 0.04 (Figure 4E).

We also quantified changes in the total NADP (i.e., NADP^+^ and NADPH) and the NADP^+^/NADPH ratio to assess intracellular energy metabolism and redox potentials, respectively.^82^ Total NADP did not differ significantly between the Au^3+^-treated and untreated control cells 1 h after irradiation (Figure 4F). After 24 h, however, the levels of total NADP in the radiation-only, Au^3+^-only, and combination treatment groups were 57%, 35%, and 76% lower, respectively, than that in the untreated control group (Figure 4F). Most dramatically, the NADP^+^/NADPH ratio in the combination treatment group 24 h after irradiation was 2.8 times larger than that in the radiation-only group and 3.0 times larger than that in the Au^3+^-only group, indicating a severe metabolic deficit (Figure 4G). Interestingly, the NADP^+^/NADPH ratios of the cells receiving only radiation or Au^3+^ were approximately 2-fold higher than that of the untreated control cells, which suggests that Au^3+^ treatment has an adverse effect on the metabolism of pancreatic cancer cells.

Finally, a thiobarbituric acid reactive substance (TBARS) assay revealed an approximately 2-fold higher level of radiation-induced peroxidation products in cells treated with the combination of Au^3+^ and radiation versus those treated with radiation alone; cells treated with Au^3+^ alone showed no induction of peroxidation product formation (Figure S7a).

Together, these findings suggest that *in situ* gold biomineralization can cause a significant radiosensitization of pancreatic cancer cells that is predominantly associated with the disruption of DNA repair, dysregulation of metabolism, and breakdown of lipids. Despite the high level of intranuclear fluorescence due to GNC formation (as shown in Figure 1D), we found no evidence of increased DNA damage due to double strand break formation.

### Metabolomic and Lipidomic Interrogation of Molecular Mechanisms of Radiosensitization by *in Situ* GNC Biomineralization

**Figure 5:**
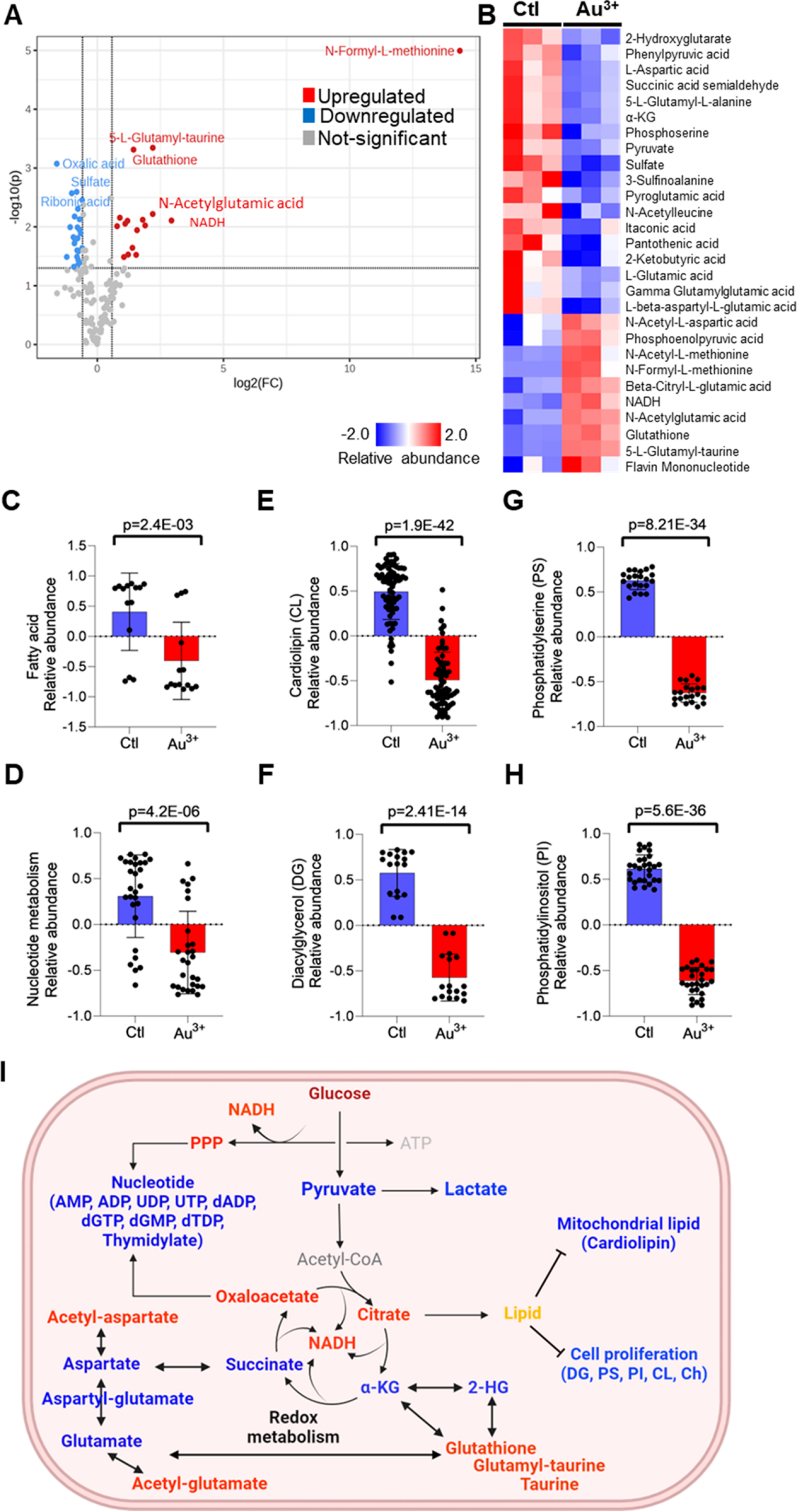
GNC biomineralization confers radiosensitization by modulating oxidative cancer metabolism and nucleotide synthesis. (A) Volcano plot showing significantly up- or downregulated metabolites in PANC1 cells after a 24 h treatment with 0.20 mM Au^3+^ compared to the untreated control (Au^3+^/Ctl). (B) Heatmap showing the relative abundance of metabolites involved in the TCA cycle and its antipleuritic networks in PANC1 cells treated with 0.20 mM Au^3+^ relative to the untreated control. Levels of fatty acid (C), nucleotide (D), cardiolipin (CL) (*n* = 75) (E), diacylglycerol (DG) (F), phosphatidylserine (PS) (G), and phosphatidylinositol (PI) (H) in Au^3+^ treated and control PANC1 cells. (I) Metabolic perturbation through redox, oxidative, energy metabolism, and cell proliferation networks in PANC1 cells treated with 0.20 mM Au^3+^ compared to the control. Red color represents elevated abundance; blue color represents reduced abundance; gray color represents no changes; black color represents a biological process. All bar graph data are presented as mean values ± standard deviation.

The specific mechanisms of the metabolic conversion of Au^3+^ to Au^0^ are not well established; however, a number of previous studies indicated the involvement of ROS/RNS,^53,56−59,61^ NADH dehydrogenase flavoprotein 2 and quinone oxidoreductase-like protein,^57^ and glutamate,^59^ as well as several proteins that bind cations, energetic metabolites, or nucleotides.^62,65,83^ If the formation of GNPs from gold ions were to disrupt the relative abundance of those biomolecules, this could enhance radiosensitization, complementing the sensitization afforded by the intracellular biosynthesis of gold particles. To explore the hypothesis that GNC biomineralization could enhance radiosensitization by modulating cancer cell metabolism and also to uncover potential biological mechanisms of gold biomineralization, we treated PANC1 cells with Au^3+^ without radiation using untreated cells as a control and performed global metabolomic and lipidomic profiling using ultrahigh pressure liquid chromatography coupled to ultrahigh resolution mass spectrometry (UHPLC-HRMS). Au^3+^-treated cells had a metabolite profile distinct from untreated cells (Figure 5B). Differential analysis revealed that Au^3+^ treatment significantly perturbed redox metabolism including NADH metabolism, glutathione metabolism, and the tricarboxylic acid (TCA) cycle (Figure 5A,B). The connection between these changes in metabolite abundance and cellular gold biomineralization were reported in the literature. Redox metabolism such as ROS/RNS, glutamate, and NADH were directly indicated as involved in cellular biomineralization.^53,56−59,61^ Additionally, many of these metabolites were shown to reduce gold ions directly through benchtop synthesis (NADH, glutathione, and citrate).^84−86^

Furthermore, Au^3+^ treatment significantly reduced fatty acid metabolism (Figures 5C and S7c) and nucleotide metabolism (Figures 5D and S7d). We also observed differential modulation of the central carbon network including glycolysis and pentose phosphate pathways (Figure S7e). The central carbon metabolic network such as the TCA cycle, NADH, and the pentose phosphate pathway (PPP) are intricately related to oxidative stress.^87^ Together, we observed distinct perturbation in central carbon metabolism that may be associated with an imbalance of cellular redox status in Au^3+^-treated cells. Additionally, through lipidomics, we observed that Au^3+^ treatment modulated the global lipid profile of PANC1 cells (Figure S7f). Lipid ontology analysis revealed significant reduction of cardiolipins (CLs) (Figures 5E and S7g). Since CLs are associated with protein complexes of the mitochondrial electron transport system (ETS), reduction of CL levels can lead to instability and impaired function of ETS and potentially result in electron leakage and impaired mitochondrial bioenergetics and increase production of ROS.^88^ This observed perturbation of CL prompts the hypothesis that the anticancer mechanism of GNC biomineralization could involve positive modulation of mitochondrial ROS production. Strikingly, Au^3+^ treatment decreased lipids known to be associated with signaling pathways for cell proliferation including diacylglycerol (DG) (Figures 5F and S7h), phosphatidylserine (PS) (Figures 5G and S7i), and phosphatidylinositol (PI) (Figures 5H and S7j).^89^ Overall, these findings identify potential mechanisms underlying gold biomineralization in mammalian cells as well as GNC-mediated radiosensitization, with effects on oxidative stress and disruption of the cell cycle highlighted as recurring themes (Figure 5I).

### Toxicity Assessment and Biodistribution

Toxicity was assessed in mice with PANC1 tumor xenografts 48 h after intratumoral injection of Au^3+^ in 20 μL of PBS. Histopathological evaluation revealed similar systemic immune stimulation in all groups of mice: mice with Au^3+^ treated tumor and mice with sham (PBS) treated tumor (Table S1). Both groups had comparable lesions of lymphocytic hyperplasia, plasmacytosis, and histiocytosis of major lymphoid tissues, mesenteric lymph nodes, and spleen. In addition, all mice from both groups had moderate to marked lymphohistiocytic inflammation in the subcutaneous tissue around the tumor. It was noticed that one of the 4 mice from Au^3+^ tumor-treated mice had intravascular tumor cell metastasis in the subcutaneous tissue near the tumor and focal subacute pyogranulomatous inflammation intermixed with atypical tumor-like cells into the mesentery. However, this and all other observed histopathologic lesions are subacute to chronic, which are older than 7 days, while Au^3+^ treatment was administered only 48 h prior to euthanasia and collection of tissue samples. Therefore, the observed local peritumoral and systemic immune inflammatory reactions were induced by the subcutaneous xenograft tumors with no significant difference between Au^3+^ treated and nontreated mice, which would support no toxicity of Au^3+^ treatment in these mice. Blood chemistry and hematology panels revealed no major differences between the Au^3+^-treated and sham treatment mice (Figure S8). The livers, gallbladders, lungs, kidneys, esophagi, hearts, skeletal muscles, aortas, and thymuses of the Au^3+^-treated mice and untreated mice did not differ significantly (Figure S9). However, Au^3+^-treated mice had a mildly higher serum globulin level and moderately higher white blood cell count (6.54 × 10^3^) in comparison with untreated mice (3.98 × 10^3^); these changes were not statistically significant (*P* = 0.4554). Of the white blood cells, both neutrophils (*P* = 0.0452) and lymphocytes (*P* = 0.4116) where higher in Au^3+^-treated mice (Figure S8). These results are consistent with the histopathological findings of lymphocytic hyperplasia in multiple lymphoid tissues (usually considered of little clinical significance)^90^ and a compensatory increased extramedullary hematopoiesis in the spleen of these mice. Although this study could not accurately evaluate the toxicity of Au^3+^ treatment of tumors because of the local and systemic immune reaction induced by xenograft tumor cells, these data show no significant histopathologic differences between Au^3+^ treated and untreated mice, which suggest a minimal or no toxicity in mice with Au^3+^ treated tumors. Our plan is to carry out toxicity studies in normal mice next to eliminate confounding factors due to tumor presence and to complement the current studies.

**Figure 6:**
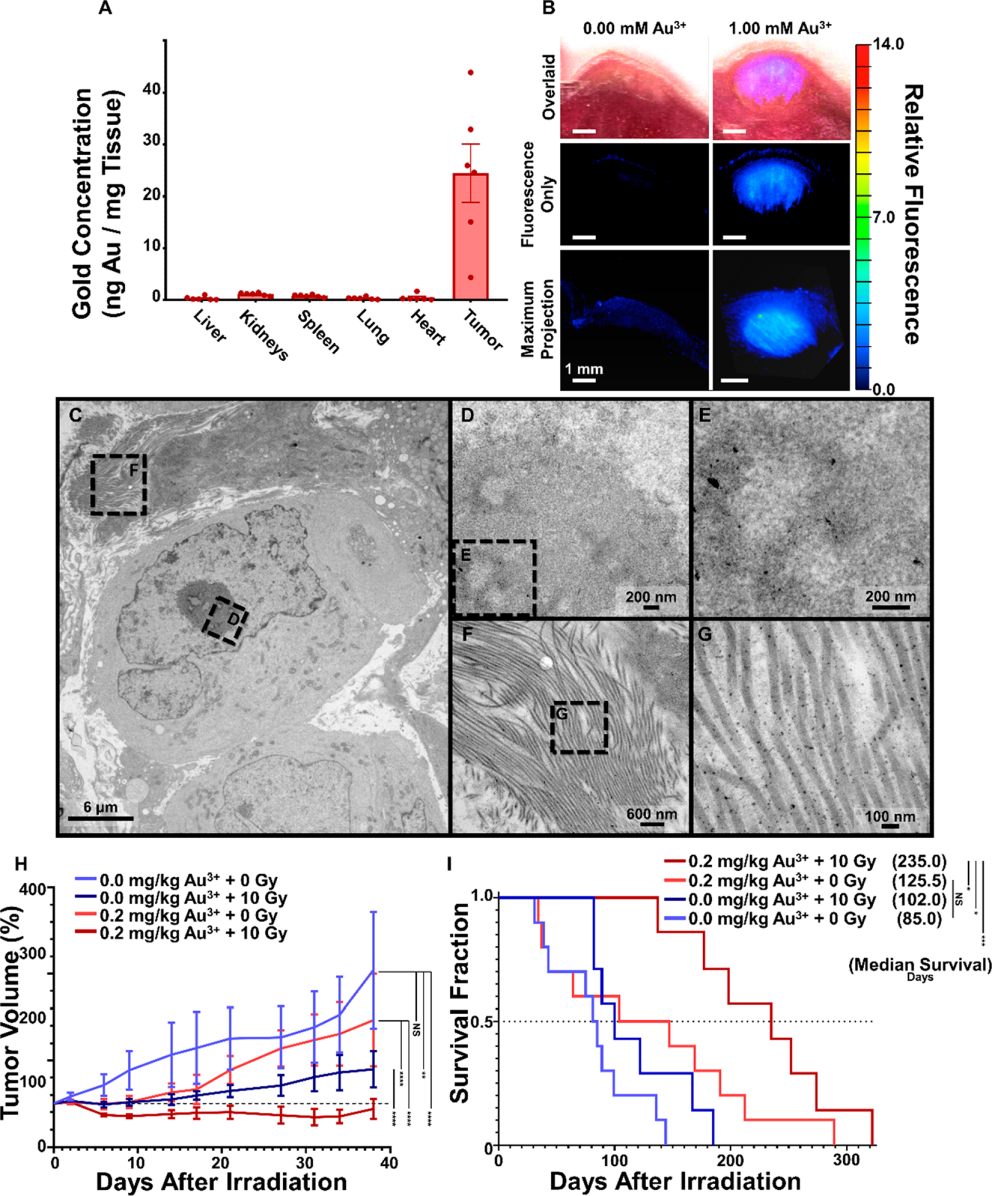
Intratumoral GNC biodistribution, biomineralization, and therapeutic radiosensitization effects. (A) ICP-MS analyses of gold content in the livers, kidneys, spleens, lungs, hearts, and tumors collected from nu/nu mice with left hind flank PANC1 tumor xenografts 48 h after intratumoral injections of 1.00 mM Au^3+^ in 20 μL of PBS. (B) Fluorescence (_ex_555 nm/_em_620 nm) and brightfield cryo-tomographic images (Emit Xerra) of PANC1 tumor xenografts 48 h after intratumoral injection of either 0.00 or 1.00 mM Au^3+^ in 20 μL of PBS. (C–G) TEM images of a PANC1 tumor xenograft harvested 48 h after intratumoral injection of 1.0 mM Au^3+^ in PBS (C), with magnified views of the nucleoli (D) and GNCs therein (E) as well as magnified views of collagen fibers (F) and GNPs therein (G). (H, I) Normalized tumor volume measurements (H) and survival fractions (I) over time for different treatments with Au^3+^ (0.0 or 0.2 mg/kg) and radiation (0 or 10 Gy). ^NS^*P* > 0.05, *P < 0.05, **P < 0.01, ***P < 0.001, and ****P < 0.0001.

Gold biodistribution was assessed by ICP-MS in PANC1 tumor xenografts 48 h after treatment with 1.0 mM Au^3+^, revealing that the amount of gold in the tumor at that time point was more than 10 times that in the liver, kidneys, spleen, lung, or heart (Figure 6A). Cryo-fluorescence tomography showed a fluorescence from GNCs in the Au^3+^-treated group that was absent in the untreated control group (Figure 6B). GNC fluorescence across multiple tumor cross sections did not fluctuate significantly, indicating a uniform distribution of GNCs in the tumor (Figure S10a,b).

We chose the 48 h time point based on previously published evaluations of temporal distribution and intratumoral clearance of Au^3+^ ions in subcutaneous murine xenograft tumor models^58,65^ that used GNC fluorescence as a tool to quantify these processes. These studies showed that the initiation of GNC formation occurs soon after Au^3+^ administration, peaking at ∼24–48 h and, then, clearing at 7 days after the treatment. Further, our *in vitro* cell studies showed that GNC formation is saturated at ∼24 h in PANC1 cells (Figure 2C). Therefore, we proceeded with the 48 h time point because it was identified as the longest time required to achieve the maximum GNC fluorescence.^58,65^ We would like to note that radiolabeling strategies for assessment of *in vivo* biodistribution that were reported for prefabricated GNP tracking^91−93^ cannot be employed for tracking Au^3+^ ions. However, previously reported use of a radioactive gold isotope ^198^Au could provide an opportunity to use nuclear imaging for *in vivo* monitoring of the *in situ* biomineralization.^94^

TEM revealed particles of 3.5 ± 2.0 nm (*n* = 156 particles) in cellular nuclei (Figures 6C–E and S10c–e,h); these sizes were similar to those observed *in vitro* (i.e., 3.1 ± 1.8 nm) (Figure S2a). Further, larger GNPs of 10.3 ± 2.2 nm (*n* = 259) were detected along the collagen fibers (Figures 6F,G and S10f,g,i), which is consistent with reports of benchtop GNP synthesis mediated by the presence of collagen.^95−97^ Thus, our TEM studies revealed that gold biomineralization *in vivo* can also occur outside cancer cells (i.e., along collagen fibers); however, the intranuclear GNC formation *in vivo* was similar to that *in vitro*. Overall, the biodistribution analysis showed that *in situ* gold biomineralization enables a strong localization of GNCs and/or GNPs within the tumor relative to nontarget sites that is ideal for tumor-specific radiotherapy enhancement. Further, these *in situ* formed nanoparticles have sizes which are consistent with an efficient renal clearance.^94,98^

### Radiosensitization *in Vivo*

We used a tumor regrowth delay study and terminal survival analysis to determine radiosensitization *in vivo* in pancreatic tumor bearing mice receiving intratumoral injections of either PBS without radiation (*n* = 10), PBS with radiation (*n* = 9), Au^3+^ without radiation (*n* = 10), or Au^3+^ with radiation (*n* = 10). Tumor regrowth across a 38-day period following the treatments showed that the negative control, Au^3+^-only, and radiation-only groups had tumor doubling times of 21, 24, and 40 days, respectively. However, the tumors in the group treated with Au^3+^ and radiation did not exhibit a statistically significant change in volume (*P* = 0.611), indicating that the treatment effectively halted tumor growth (Figure 6H).

To compare tumor burden-induced mortality between treatment groups, we performed terminal survival analyses to identify and exclude events associated with nontarget competitive risks (i.e., not associated with tumor burden). These analyses indicated five mortality events that were not related to tumor burden and were thus excluded from the survival study (Supporting Materials and Figure S11). Survival analyses of mice whose mortality events were associated with tumor burden revealed that the median survival duration of the Au^3+^-plus-radiation group (*n* = 7; 235 days;) was significantly longer than those of the nonirradiated control group (*n* = 10; 83 days), radiation-only group (*n* = 7; 102 days), and Au^3+^-only group (*n* = 10; 125.5 days) (Figure 6H). There were no statistically significant differences in survival between the control groups, indicating that Au^3+^ treatment or radiation alone were not able to provide significant relief.

## Conclusions

Our findings demonstrate cancer radiosensitization through biosynthesis of GNCs and GNPs *in vitro* and *in vivo*. We found that intracellular gold biomineralization occurs with higher efficiency in cancerous versus noncancerous pancreatic cells and is associated with a strong nuclear localization of fluorescent GNCs, especially into nucleoli. We also showed that *in situ* biomineralized GNCs and GNPs radiosensitize pancreatic cancer cells by dysregulating DNA repair and cell metabolism and by accentuating peroxidation. We demonstrated that a radiosensitization strategy that relies on the delivery of an ultimately small precursor, Au^3+^ ion, for the *in situ* formation of GNCs and GNPs can significantly increase the efficiency of radiotherapy and provide survival benefits. This elegantly simple approach takes advantage of complex biological machinery for intranuclear cancer targeting and does not require the sophisticated and potentially expensive synthesis of targeted nanoparticles. Interestingly, previously published reports demonstrated the possibility to control the size and morphology of *in situ* gold particle synthesis for application in photothermal therapy.^63,65^ In one study, a polyethylene glycol (PEG) coaggregation with gold ions was used to promote the formation of larger GNPs to increase the photothermal effect.^65^ In another study, the authors were able to promote the formation of gold nanoribbons by adding prefabricated GNPs to cells cotreated with gold ions.^63^ The strategy utilizing a PEG nanovector for gold ion delivery could be more clinically relevant because it relies on delivery of a single entity whereas the other approach requires two components wherein the timing between their delivery is an essential component of the reaction’s control. These studies indicate the feasibility of an engineering approach toward optimization of gold biomineralization for a specific biomedical application.

By obviating the need for systemic delivery, in situ gold biomineralization overcomes the challenge of evading reticuloendothelial capture of nanoparticles administered via the bloodstream and also avoids the need for nuclear transport moieties through the innate nuclear localization in cancer cells. Furthermore, our data show that this strategy can overcome delivery barriers associated with a dense tumor environment and cellular cytoplasmic and nuclear membranes. These studies could catalyze the future clinical translation of gold biomineralization for cancer radiotherapy.

## Methods

### Cell Culture

PANC-1 and Mia-PaCa-2 cells were cultured according to ATCC guidelines in DMEM with 10% v/v FBS and 1× penicillin–streptomycin with the addition of 2.5% horse serum to the Mia-PaCa-2 cell media. HPDE cells were grown in keratinocyte serum-free complete media supplemented with 1× antibiotic–antimycotic. All cells were incubated under standard culture conditions (i.e., ∼95% humidity, 5% CO_2_, 37 °C, normal pH). Cells were passaged 2 or 3 times weekly using 0.25% trypsin-EDTA to prevent them from becoming overly confluent. In lifting HPDE cells, a trypsin inhibitor was used to quench trypsin activity before the cells were passaged to fresh culture dishes.

### Intracellular GNC Biomineralization

A small volume (<1% of the total cell culture volume) of sterile filtered (using a 0.22 μm filter) chloroauric acid at a concentration of 50 or 100 mM (i.e., Au^3+^ mM) in Milli-Q water (18.2 MΩ) was added directly to the culture media. In a typical experiment, cells were grown in a T-75 cell culture flask up to 70–90% confluency ((6–8) × 10^6^ cells) before the treatment. Cells were incubated with Au^3+^ under normal cell culture conditions unless otherwise indicated. Treatments with supplementary cationic salts were administered in a sterile media in combination with the gold ion treatment. Sterile cell culture techniques were used throughout.

### Confocal Microscopy

Cells were plated at 60–70% confluency in 8-well chambered coverglass slides (Lab-Tek) and allowed 24 h to adhere before treatment with Au^3+^. Cells were imaged using an SP8 Laser Scanning Confocal Microscope (Leica). A 561 nm excitation laser and a 610 ± 20 nm emission filter were used to detect GNC fluorescence using an avalanche photodiode photomultiplier tube detector. For the imaging of unstained cells, cells were treated with 1.00 mM Au^3+^ and allowed to incubate for 24 h before imaging. For the imaging of cells with nuclear staining, PANC1 cells were stained with Hoechst 33342 according to the manufacturer’s protocol using Hoechst solution in PBS at 1:2000 dilution for 30 min followed by imaging within 30 min. The total nuclear GNC fluorescence per cell was quantified using Imaris software and the blue Hoechst 33342 channel to demarcate cell nuclei. For longitudinal imaging, we used an environmental imaging chamber attachment maintained at normal cell incubating conditions (i.e., ∼95% humidity, 5% CO_2_, 37 °C), and images were collected once every 10 min for 20 h.

### TEM

Samples were fixed with a solution containing 3% glutaraldehyde plus 2% paraformaldehyde in 0.1 M cacodylate buffer, pH 7.3, then washed in 0.1 M sodium cacodylate buffer and treated with 0.1% Millipore-filtered cacodylate buffered tannic acid, postfixed with 1% buffered osmium tetroxide, and stained en bloc with 1% Millipore-filtered uranyl acetate. The samples were dehydrated in increasing concentrations of ethanol, infiltrated, and embedded in LX-112 medium. The samples were polymerized in a 60 °C oven for approximately 3 days. Ultrathin sections were cut in an Ultracut microtome (Leica), stained with uranyl acetate and lead citrate, and examined using a JEM 1010 transmission electron microscope (JEOL) at an accelerating voltage of 80 kV. Digital images were obtained using an imaging system from Advanced Microscopy Techniques.

### Irradiation of Cells

For X-ray irradiation of cells, the plastic lids of irradiated plates were temporarily replaced with a clean parafilm seal in a sterile biosafety cabinet. The parafilm-sealed plates were placed onto the bed of an XRAD SmART small animal irradiator (Precision XRay Inc.). A scout computed tomography (CT) scan was used to center the sample at the X-ray beam’s isocenter within the field of view. A 4 cm × 4 cm square collimator was placed over the CT tube, and a 0.3 mm copper treatment filter was inserted. X-ray doses ranging from 2 to 10 Gy were delivered using an anterior–posterior treatment plan with a voltage of 225 kV and the current set to 20 mA.

### MTS Assays

A CellTiter 96 AQueous Non-Radioactive Cell Proliferation Assay kit (Promega) was used according to the manufacturer’s instructions. A detailed description is provided in the Supporting Information.

### JC-1 Assay

JC-1 mitochondrial membrane potential stain (Life Technologies) was prepared and used according to the manufacturer’s instructions. A detailed description is provided in the Supporting Information.

### AO/PI Live–Dead Assay

Cells were seeded in T25 tissue culture flasks and incubated for approximately 24 h before Au^3+^ biomineralization treatments with chloroauric acid at final concentrations of 0.00, 0.10, or 0.20 mM Au. Cells were incubated with the Au^3+^ ions overnight followed by cell collection via trypsinization. Finally, cells were resuspended in PBS and briefly admixed with an Nexcelom AO/PI dye assay kit at a 1:1 (v/v) ratio immediately before 20 μL was dispensed to Cellometer Cell Counting Chambers for quantitation of viability using a Cellometer Auto 2000 Cell Viability Counter (Nexcelom) per the manufacturer’s instructions.^99^

### Flow Cytometry

Cells were seeded in T75 tissue culture flasks at uniform density (60% confluency) and incubated for approximately 24 h before treatment with 0.20 mM Au^3+^ for 24 h; untreated cells were used as a control. After the treatment, the cells were washed with PBS, detached via trypsinization, washed with PBS, and stained with Hoechst 33342 according to the manufacturer’s instructions. Then, the cells were washed with cold PBS (4 °C) by centrifugation (160*g* for 6 min); the cell pellet was resuspended in 500 μL of cold PBS, and the cells were immediately analyzed by flow cytometry using a 5-laser, 18-color LSRFortessa X-20 Analyzer (BD FACS Calibur, CA, USA). For each sample, 10,000 events were collected. The data were analyzed using FlowJo_v10.9.0 Software. Cell populations were gated using plots of side-scatter versus Hoechst intensity (355 nm excitation laser, 450/50 nm filter) to exclude debris, and histograms of the GNC channel intensities (561 nm excitation laser, 610/20 nm filter) were analyzed. The percentage of GNC positive cells was determined as the percentage of events in the treatment group that have a greater GNC intensity than the median plus robust σ of the untreated control, using methodology described in refs (100 and 101).

### Clonogenic Survival Assay

PANC1 cells at approximately 70% confluency in T-75 culture flasks were treated with either 0.00 or 0.20 mM Au^3+^ and incubated for 24 h. The cells were then lifted with trypsinization, passaged to 35 mm tissue culture-treated dishes at concentrations ranging from 50 to 8,000 cells/plate (*n* = 6 replicates), and allowed to settle for 30–60 min before X-ray irradiation with 0, 2, 4, or 6 Gy. After irradiation, the cells were incubated in cell culture media for 14–21 days to form colonies. The colonies were washed with PBS and fixed with a 1:7 mixture of acetic acid and methanol. After fixation, the colonies were briefly stained with a 0.5% (g/g) crystal violet solution and washed with PBS, and the plates were placed upside-down to air-dry for 2 days. The colonies were imaged and manually counted using a UVP GelSolo gel documentation system (Analytik Jena). Plating efficiency and survival fractions were calculated using the procedure described by Franken et al.^102^

### γ-H2AX Foci Staining and Quantification

An Alexa Fluor 488-labeled γ-H2AX antibody (Fisher Scientific) was used to quantify double-stranded DNA breaks in cells treated with Au^3+^ and/or radiation. Cells were seeded in LabTek culture plate slides (#1.5) for 24 h to adhere before treatment with Au^3+^ and/or radiation. Following treatment, cells were washed with PBS three times, fixed with cold methanol (−20 °C) for no more than 30 min, and then washed three times with cold PBS (4 °C) containing 5% FBS. An Alexa Fluor-linked antibody in cold PBS with 5% FBS (1:2000 dilution) was added to the cells. Following overnight incubation at 4 °C, the cells were stained with Hoechst 33342 and imaged using an SP8 Laser Scanning Confocal Microscope (Leica) with a 488 nm excitation laser and a 610 ± 20 nm emission filter.

### NADP/NADPH Quantification Assay

The NADP/NADPH quantification assay (MilliporeSigma) was performed according to the manufacturer’s instructions. A detailed description is provided in the Supporting Information.

### TBARS Assay

The TBARS assay (MilliporeSigma) was performed according to the manufacturer’s instructions. A detailed description is provided in the Supporting Information.

### Nontargeted Metabolomics

To determine the relative abundance of polar metabolites in cell samples, extracts were prepared and analyzed by ultrahigh resolution mass spectrometry (HRMS). Metabolites were extracted using ice-cold 0.1% ammonium hydroxide in 80/20 (v/v) methanol/water. Extracts were centrifuged at 17,000*g* for 5 min at 4 °C, and supernatants were transferred to clean tubes, followed by evaporation to dryness under nitrogen. Dried extracts were reconstituted in deionized water, and 5 μL was injected for analysis by ion chromatography (IC)-MS. IC mobile phase A (MPA; weak) was water, and mobile phase B (MPB; strong) was water containing 100 mM KOH. A Thermo Scientific Dionex ICS-5000+ system included a Thermo IonPac AS11 column (4 μm particle size, 250 × 2 mm) with the column compartment kept at 30 °C. The autosampler tray was chilled to 4 °C. The mobile phase flow rate was 350 μL/min, and a gradient from 1 to 100 mM KOH was used. The total run time was 60 min. To assist the desolvation for better sensitivity, methanol was delivered by an external pump and combined with the eluent via a low dead volume mixing tee. Data were acquired using a Thermo Orbitrap Fusion Tribrid Mass Spectrometer under ESI negative ionization mode at a resolution of 240,000.

### Nontargeted Lipidomics

To determine the relative abundance of lipid in PANC1 cells, extracts were prepared and analyzed by the high-resolution mass spectrometry-based lipidomics at the MD Anderson Cancer Center Metabolomics Core Facility. Briefly, to each cell sample, 200 μL of extraction solution containing 2% Avanti SPLASH LIPIDOMIX Mass Spec Standard and 1% 10 mM butylated hydroxytoluene in ethanol was added, and the tubes were vortexed 10 min. The tubes sat in ice for 10 min and were centrifuged at 13,300 rpm for 10 min at 4 °C. The supernatant was transferred to a glass autosampler vial, and the injection volume was 10 μL. Mobile phase A (MPA) was 40:60 acetonitrile:water with 0.1% formic acid and 10 mM ammonium formate. Mobile phase B (MPB) was 90:9:1 isopropanol:acetonitrile:water with 0.1% formic acid and 10 mM ammonium formate. The chromatographic method included a Thermo Fisher Scientific Accucore C30 column (2.6 μm, 150 × 2.1 mm) maintained at 40 °C, autosampler tray chilled at 8 °C, a mobile phase flow rate of 0.200 mL/min, and a gradient elution program as follows: 0–3 min, 30% MPB; 3–13 min, 30–43% MPB; 13.1–33 min, 50–70% MPB; 48–55 min, 99% MPB; 55.1–60 min, 30% MPB.

A Thermo Fisher Scientific Orbitrap Fusion Lumos Tribrid mass spectrometer with heated electrospray ionization source was operated in data dependent acquisition mode, in both positive and negative ionization modes, with scan ranges of 150–827 and 825–1500 *m*/*z*. An Orbitrap resolution of 120,000 (fwhm) was used for MS1 acquisition and spray voltages of 3,600 and −2,900 V were used for positive and negative ionization modes, respectively. Vaporizer and ion transfer tube temperatures were set at 275 and 300 °C, respectively. The sheath, auxiliary, and sweep gas pressures were 35, 10, and 0 (arbitrary units), respectively. For MS^2^ and MS^3^ fragmentation, a hybridized HCD/CID approach was used. Each sample was analyzed using four injections making use of the two aforementioned scan ranges, in both ionization modes. Data were analyzed using Thermo Scientific LipidSearch software (version 5.0) and R scripts written in house.

### Xenograft Implantation and Gold Treatment Administration

PANC-1 cells determined to be negative for mycoplasma by MD Anderson’s Cytogenetics and Cell Authentication Core were used. All animal protocols were approved by MD Anderson’s Institutional Animal Care and Use Committee. For xenograft implantation, approximately 2 × 10^6^ cells/100 μL sterile filtered PBS were admixed with an equivalent volume of Matrigel, resulting in approximately 1 × 10^6^ cells/100 μL injection suspension. Fifty microliters of the cell suspension (∼0.5 × 10^6^ cells) was injected subcutaneously into the left hind flanks of nu/nu mice under isoflurane anesthesia using a 30G needle. Intratumoral injections of Au^3+^ were performed when tumors reached approximately 70 mm^3^ (∼50 days after cell implantation). The mice were given 20 μL intratumoral injections of either sterile filtered phosphate-buffered saline (PBS) or 1.00 mM Au^3+^ in PBS (i.e., ∼0.2 mg Au/kg mouse weight). We chose direct intratumoral injection as the delivery approach because of the hypovascular nature of pancreatic cancer and as a means of penetrating stroma without disrupting it. Furthermore, recent clinical studies showed that intratumorally administered TNFerade^103^ and functionalized hafnium oxide nanoparticles^4^ are well-tolerated and feasible options in the treatment of pancreatic ductal adenocarcinoma.

### Histopathological Evaluation

Xenograft tumor-bearing mice were euthanized 48 h after intratumoral Au^3+^ treatment, and tissue samples from the liver, kidney, lung, trachea, heart, aorta, spleen, mesenteric lymph node, thymus, esophagus, and tumor xenograft were fixed in 10% neutral buffered formalin solution for 72 h. Formalin-fixed tissues were embedded in paraffin blocks, sectioned, and stained with hematoxylin and eosin (H&E) for histopathological evaluation by a board-certified veterinary pathologist. Microscopic examination of histologic sections was performed using an Olympus BX41 microscope coupled to a Leica DFC495 camera. Histologic changes or lesions were recorded and scored for extent and severity.

### Blood Chemistry and Hematology Analysis

Blood samples were collected immediately after euthanasia via cardiocentesis into microtubes containing either EDTA for the hematology analysis or a serum separator for the blood chemistry analysis. Within 4 h of sample collection, complete blood counts were analyzed with the ADVIA 120 Hematology System (Siemens). Blood chemistry, including serum levels of albumin, alkaline phosphatase, alanine transaminase, aspartate transaminase, blood urea nitrogen, creatinine, globulin, and total protein, was analyzed using the COBAS INTEGRA 400 Plus system (Roche).

### Quantification of Gold

The elemental gold content in the *in vitro* and *in vivo* samples treated with chloroauric acid was quantified using a NexION 2000B ICP-MS system (PerkinElmer) after sample preparation with the hot plate dissolution technique using TraceMetal grade concentrated acids. The tissue samples were vacuum-dried for >48 h, accurately weighed, and then solubilized by treatment with 67–69% nitric acid (Fisher A467-1) and 35–38% hydrochloric acid (Fisher A466-1) under a fume hood. First, 2.5 mL of nitric acid was added to samples for 24 h at room temperature. Then, 0.5 mL of hydrochloric acid was added, and the vial was closed using a screw cap with a preslit polytetrafluoroethylene liner and kept on top of a hot plate at 90 °C for 96–480 h, until the liquid inside became completely clear. The vial was cooled to room temperature, and the cap was carefully removed. The open vial was placed on top of a hot plate at 120 °C until the volume reduced to approximately 0.5 mL. The residue was diluted with a solution of 1% nitric acid and 3% hydrochloric acid in deionized water to the final volume of 10 mL. The resulting solution was then filtered through 0.2 μm glass microfiber syringe filters into a 15 mL PP Eppendorf tube. For ICP-MS, a glass cyclonic sample introduction system was used along with a SMARTintro sample introduction cassette (Blue) with a 2.0 mm fixed injector and a MEINHARD concentric nebulizer. Settings used for ICP-MS samples were as follows: nebulizer flow rate, 1.14 L/min; plasma gas flow rate, 18 L/min; radiofrequency power, 1600 W; dwell time, 30 μs; helium reaction gas flow rate, 1.5 mL/min. Each sample was followed by a blank to eliminate any measurement inconsistencies due to gold carryover (i.e., instrument memory effect). Before the analysis, the instrument was optimized using the automated SmartTune procedure targeting maximum gold sensitivity (Syngistix ICP-MS software, v.2.5.1904.21469). Gold concentrations were calculated from a regression equation built with a set of standards with concentrations of 1 × 10^–10^ to 1 × 10^–4^ g/L prepared by diluting the gold primary standard (TraceCERT, 1000 mg/L). A 0.10 μg/L Ga^3+^ internal standard was added to the blank, all calibration standards, and all samples. The Ga^3+^ standard was prepared by diluting the gallium primary standard (1000 mg/L, Specpure) in a polypropylene volumetric flask to the desired volume with 2% hydrochloric acid in deionized water.

### Xerra Cryo-Tomographic Fluorescence Imaging

All mice were handled in accordance with MD Anderson’s Institutional Animal Care and Use Committee guidelines. Immediately after euthanasia, mice were frozen by dipping them head-first into hexane cooled with dry ice and then stored at −20 °C for no more than 2 weeks before they were embedded in optimum cutting temperature compound using a special mold designed for Xerra imaging. The embedded mice were affixed to the Xerra stage using a screw mechanism. The Xerra imaging protocol included both white light and fluorescence imaging (_ex_555 nm/_em_620 nm) with a 50 μm slice thickness. The imaging data were reconstructed, normalized, and extracted using the Emit software program. Pixel fluorescence intensity values for tumor slices were analyzed using ImageJ, with the intensity values normalized by the corresponding exposure times.

### Irradiation of Tumor Xenografts

Radiotherapy was administered 48 h after intratumoral injections of either 20 μL sterile filtered PBS (negative control; *n* = 17) or 1.00 mM Au^3+^ in PBS (*n* = 20). A single dose of 10 Gy was administered to mice treated with either PBS (radiation only; *n* = 7) or Au^3+^ (radiation plus Au^3+^; *n* = 10); 10 mice were used as an untreated control (i.e., PBS injection only) group, and 10 mice were used as a Au^3+^ treatment group (i.e., Au^3+^ injection only). Mice were anesthetized using 2% isoflurane and placed onto the bed of an XRAD SmART small animal irradiator (Precision XRay Inc.). A scout CT scan was used to center the tumor at the beam isocenter within the field of view. A 1.5 cm collimator was placed over the CT tube, and a 0.3 mm copper treatment filter was inserted. A 10 Gy dose was delivered using an anterior–posterior/posterior–anterior treatment plan. The anterior–posterior beam time was 71 s, and the posterior–anterior beam time was 78 s. The voltage used was 225 kV, and the current was set to 20 mA. All mice recovered in a warm clean cage after irradiation.

### Weight and Tumor Volume Measurements

Following tumor implantation, mouse weight and tumor volume were assessed weekly using a gram scale and caliper measurements, respectively. After X-ray irradiation, tumor size and mouse weight were collected twice a week. Tumor volume was calculated using the equation *V* = *L* × *W* × *W*, where *L* is the larger dimension and *W* is the smaller. Mice were euthanized with CO_2_ asphyxiation followed by cervical dislocation if they had ≥20% weight loss from baseline, a tumor burden exceeding 2 cm in any dimension, or any other major health issues.

## Supporting Information Available

Optimization of treatment conditions for intracellular gold nanocluster biomineralization (S1.1.), competitive risk events in survival studies (S1.2.), materials used (S1.3.), and detailed kit assay methods (S1.4.); confocal fluorescence images; analysis of cellular TEM and optical properties of albumin-coated GNCs; gold nanoparticle biosynthesis and cell viability; cell viability and ROS induction by physiological cationic ions; uptake of gold ions; formation of peroxidation products; biochemistry and hematology analysis; histology of normal organs; intratumoral GNC biomineralization; analysis of competitive risk events; assay calibration plots; histopathology evaluation results (PDF)
Movie S1: gold nanocluster (GNC) formation inside live PANC1 cells over a period of 20 h; acquired every 10 min using confocal fluorescence microscopy with a 561 nm excitation laser and a 610 ± 20 nm emission filter to detect GNC fluorescence (red); under standard culture conditions (i.e., ∼95% humidity, 5% CO_2_, 37 °C, normal pH) (MP4)

## Notes

A preprint version of these studies was submitted to the ChemRxiv server.^104^

## Abbreviations

GNP: gold nanoparticle
GNC: gold nanocluster
Au^3+^: ionic gold from chloroauric acid
TEM: transmission electron microscopy
FBS: fetal bovine serum
ICP-MS: inductively coupled plasma mass spectrometry
JC-1: tetraethylbenzimidazolylcarbocyanine iodide
NADP: nicotinamide adenine dinucleotide phosphate
TBARS: thiobarbituric acid reactive substance
CT: computed tomography
PBS: phosphate-buffered saline
H&E: hematoxylin and eosin
UHPLC-HRMS: ultrahigh resolution mass spectrometry
