# Supplementary material for: Intratumoral Biosynthesis of Gold Nanoclusters by Pancreatic Cancer to Overcome Delivery Barriers to Radiosensitization
Source: ACS Nano. 2024 Jan 11;18(3):1865–81. doi: 10.1021/acsnano.3c04260 (PMC10811688; doi:10.1021/acsnano.3c04260)
Supplement: Supplementary file 1 — nn3c04260_si_001.pdf [file nn3c04260_si_001.pdf]

Supporting Information: Intratumoral biosynthesis of gold nanoclusters by pancreatic cancer to overcome delivery barriers to radiosensitization

Aaron S. Schwartz-Duval,[σ] *Yuri Mackeyev,*[ψ] *Iqbal Mahmud,*[ϰ] *Philip L. Lorenzi,*[ϰ] *Mihai Gagea,*[ε] *Sunil Krishnan,*[ψ*] *and Konstantin Sokolov* [σ*]

[σ]Department of Imaging Physics, The University of Texas MD Anderson Cancer Center, 1515 Holcombe Boulevard, Houston, TX 77030, USA

[ψ]Vivian L. Smith Department of Neurosurgery, University of Texas Health Science Center, Houston, TX 77030, USA

[ϰ]Department of Bioinformatics and Computational Biology, The University of Texas MD Anderson Cancer Center, 1515 Holcombe Boulevard, Houston, TX 77030, USA

[ε]Department of Veterinary Medicine & Surgery, The University of Texas MD Anderson Cancer Center, 1515 Holcombe Boulevard, Houston, TX 77030, USA.

KEYWORDS

biomineralization, gold nanoparticles, *in situ* therapies, radiosensitization, pancreatic cancer

Appendix

Supplementary Text Related to

S1.1. Optimization of treatment conditions for intracellular gold nanocluster biomineralization.

S1.2. Competitive risk events in survival studies.

S1.3. Materials used.

S1.4. Detailed kit assay methods.

Figures. S1 – S12

Table S1

References (1 to 6)

Other Supplementary Materials for this manuscript include the following:

Movie S1

Supplementary Text

S1.1. Optimization of treatment conditions for intracellular gold nanocluster biomineralization. First, we evaluated the influence of cell secretions on *in situ* gold nanocluster (GNC) biomineralization by allowing cells to incubate in the culture media for 0–72 hours prior to treatment with Au^{3+} and found no apparent trends in nuclear fluorescence from GNC biomineralization (**Figure 2B and Figure S3b**). Then, we studied intranuclear GNC fluorescence as a function of time in cells treated with 1.00 mM Au^{3+} for 0.0–24.0 hours (**Figure 2C and Figure S3c**). The fluorescence signal appeared as early as 30 minutes from the start of the treatment, then peaked at 4 hours with an apparent decrease in signal at 20 hours that recovers at 24 hours. In the study of the dependence of intranuclear GNC fluorescence on the Au^{3+} concentration, the treatment time was fixed at 24 hours (**Figure 2D and Figure S3d**). The fluorescence did not differ significantly between cells treated with 0.05–0.10 mM Au^{3+} and untreated cells. Cells treated with 0.20–0.75 mM Au^{3+} exhibited a similar fluorescence signal that was approximately 1.7 times greater than that in cells treated with 1.00 mM Au^{3+}. We hypothesized that the decrease at the 1.00 mM dose could be associated with the reduction in number of GNCs due to formation of larger, nonfluorescent gold nanoparticles (GNPs). A similar trend in formation of larger gold particles at higher concentrations of Au^{3+} has been observed and characterized in[1].

The absorbance spectra of Au^{3+}-treated PANC1 cells in full media showed the formation of large GNPs at Au^{3+} concentrations ≥1.00 mM (**Figure S4a, b**). The combined characterization of the formation of both small fluorescent GNCs and larger GNPs in Au^{3+}-treated PANC1 cells in full growth media using Synergy HT microplate reader revealed an increase in GNC fluorescence without an evident formation of GNPs at concentrations between 0.00 and 0.75 mM Au^{3+}. At higher concentrations, GNC fluorescence decreased with a concurrent increase in absorbance from

GNPs (**Figure S4c**). The evaluation of noncancerous human pancreatic duct epithelial (HPDE) cells showed practically no formation of GNCs and a significantly lower absorbance form GNPs compared to cancerous cells, albeit the initial formation of GNPs was detected at a lower Au^{3+} concentration than in cancerous cells (**Figure S4d**). The viability of PANC1 cells assessed by 3-(4,5-dimethylthiazol-2-yl)-5-(3-carboxymethoxyphenyl)-2-(4-sulfophenyl)-2H-tetrazolium (MTS) assay was not affected by treatments of 0.0–0.20 mM Au^{3+}, but it decreased to 81 ± 2% in response to the treatment of 0.75 mM Au^{3+}; at concentrations >0.75 mM Au^{3+}, this assay was no longer viable due to an interference from a broad absorbance peak (about 550 nm) from the formation of GNPs (**Figure S4f**). To address this limitation, we used tetraethylbenzimidazolylcarbocyanine iodide (JC-1) mitochondrial depolarization and acridine orange and propidium iodide (AO/PI) live-dead assays to characterize the impact of Au^{3+} treatment on cell mitochondrial potential and viability, respectively. The JC-1 assay showed the 523/590 JC-1 fluorescence ratio to be approximately 1.2 for all treatments between 0.20 and 1.50 mM Au^{3+}, indicating that the Au^{3+} treatments in that concentration range did not considerably alter the mitochondrial membrane potential (**Figure S4h**). The AO/PI assay revealed no significant differences in viability among cells treated with 0.00–0.20 mM Au^{3+} (**Figure S4i**); however, cells treated with higher Au^{3+} concentrations would not lift from the plates via trypsinization, which prevented their assessment at those concentrations by this assay. Based on the combination of these assays we concluded that Au^{3+} treatments with concentrations ≤20 mM do not adversely affect viability of PANC1 cells.

S1.2. Competitive risk events in survival studies. Competing events in survival studies are distinct from and preclude events of interest,[S2-3] with conventions supporting the exclusion of competing

risk events that prevent the observation or occurrence of events of interest. [S3] For our survival studies, the event of interest was mortality caused by tumor burden. An initial analysis of the terminal survival studies for the combined Au^{3+} and radiation treatment group revealed a pattern of early and late mortality events separated by a long delay (**Figure S11a**), which suggests the presence of competitive risk events. This pattern was distinct from survival curves without competitive risk events, which typically have hazard rates that are either monotonically increasing, decreasing, or constant across time.[S4] Comparing the rate of mortality events across time revealed significantly distinct rates of mortality (**Figure S11b**), reflecting early (hazard 1), mixed (the long delay between mortality events), and late (hazard 2) hazard risks. We used a second derivative inflection-point test of the survival curve to identify the point in time separating the early and late risk events,[S5] which was 46 days after irradiation (**Figure S11c, d).** Analysis of tumor burden data for individual mice in the combined treatment group indicated that hazard 1 mortalities were not likely caused by tumor burden because all early mortality events were associated with tumor volumes at or below initial pre-treatment values (**Figure S11e**). Further, similar early mortality events that were not related to tumor growth were also observed in both the radiation-only treatment group (n=2) and untreated control group (n=1). Therefore, we repeated this process for all other groups (**Figure S11f-l**). Possible evidence of competing hazards, through multiple rates of mortality occurrence, was found in the non-treatment control group (**Figure S11f**); however, the inflection time-point between these potential hazards was very close to the median survival time, indicating that these hazards were evenly distributed across the duration of study (**Figure S11g, h**). We did not find sufficient evidence of competing risk within the Au^{3+}-only treatment group (**Figure S11i**); however, we did find this evidence in the radiation-only treatment group, with an inflection point between hazards at 65 days following irradiation (**Figure S11j-l**). On the

basis of these tests, we removed mice whose mortality events were not associated with tumor burden (our hazard of interest); that were from groups identified with multiple statistically distinct mortality rate groupings; and for which the calculated inflection points between hazards were separate from the median survival (*i.e.*, were non-homogeneously distributed). Using this approach, we excluded acute competing hazards that were not associated with our hazard of interest (tumor burden), enabling us to more clearly evaluate and draw comparisons between different treatment groups.[S3]

S1.3. Materials. Mia-PaCa-2 cells were obtained from MD Anderson's Cytogenetics and Cell Authentication Core. PANC-1 cells (CRL-1469), DMEM (30-2002), Dulbecco's phosphate-buffered saline (PBS) without Ca or Mg (DPBS) (30-2200), horse serum (30-2040), and 100x penicillin-streptomycin (30-2300) were purchased from ATCC. Chloroauric acid (Au^{3+}) (520918-25G), Fetal bovine serum (F0320), trypsin–ethylenediamine tetra-acetic acid solution 0.25% (T4049), dimethyl sulfoxide (D2650), perchloric acid 70% (244252), sulfuric acid 95-98% (258105), 1-butanol (360465), the NADP/NADPH quantitation kit (MAK038), the lipid peroxidation assay kit (MAK085), Matrigel (126), methanol (320390), glacial acetic acid (AX0073-9), crystal violet powder (50186559), Thiobarbituric acid reactive substance assay (MAK085) and TraceCERT 1000 mg/L inductively coupled plasma mass spectrometry (ICP-MS) Au(III) standard in hydrochloric acid (67363) were purchased from Millipore Sigma. T-75 (NC1135203) and T-175 (50809259) tissue culture flasks with vent caps, 96-well tissue culture plates (087722C), 8-well chambered coverglass slides (12565338), Alexa Fluor 488–conjugated polyclonal γ-H2AX antibody (NB100384AFC), digital calipers (50996491), keratinocyte serum-free media (17005042), borosilicate glass vials (2991361) and caps (3410044), metal-free 67-69%

nitric acid (S020101TFIF05), metal-free 35-38% hydrochloric acid (S020401TFIP05), and Specpure 1000 μg/ml gallium plasma standard solution (13869) were purchased from Fisher Scientific. The Tissue culture–treated dishes (35 mm; P659-430165) were purchased from Quality Biological Inc. The MitoProbe JC-1 assay kit (M34152), 100x antibiotic-antimycotic (15240062), Hoechst 33342 (H1399), and soybean trypsin inhibitor (17075029) were purchased from Life Technologies. The MTS assay kit (G5430) was purchased from Promega Corp. Isoflurane (sc-363629Rx) was purchased from Santa Cruz Biotechnology Inc. A compact portable scale (CB1001) for measuring mouse body weights was purchased from Braintree Scientific Inc. Sodium cacodylate buffer, paraformaldehyde for transmission electron microscopy (TEM), and glutaraldehyde fixatives were purchased from Electron Microscopy Sciences. LX-112 resin was purchased from Ladd Research Industries. Uranyl acetate and lead citrate chemicals were purchased from Electron Microscopy Sciences. The AO/PI assay kit was purchased from Nexelcom Bioscience. Syringe filters with fiberglass membranes (13 mm; 6894-1304) were purchased from VWR. Low-dose insulin syringes (0.3 cc; 26014) were purchased from EXELint. Needles (30G x .5 inch; 305106) were purchased from BD. Sterile 1-ml syringes were from Henry Schein Animal Health (060734). HPDE cells (ECA001-FP) were purchased from Kerafast.

S1.4. Detailed kit assay methods.

MTS assays. MTS and phenazine methosulfate (PMS) solutions were admixed before use according to the CellTiter 96 AQueous Non-Radioactive Cell Proliferation Assay kit instructions (Promega). To determine the effect of chloroauric acid on cell viability, we first plated cells in a clear-bottom 96-well tissue culture–treated plate at a seeding density of 20,000 cells/well in

standard cell media in accordance with the supplier's instructions. Cells were allowed to adhere and incubate for 24 hours at standard culture conditions. The cell media was then removed and replaced with 150 µl of fresh media. Fifty-microliter aliquots of chloroauric acid at concentrations of 0.00, 0.40, 0.80, 2.00, 3.00, 4.00, 6.00, and 8.00 mM Au^{3+} were added to the wells, bringing the total volume of each well to 200 µl. Then, the cells were incubated under standard culture conditions for approximately 20 hours. The background absorbance spectra were collected before adding 20 µl of the combined MTS and PMS solution to each well. The cells were incubated for 4 additional hours, and absorbance at 450 nm was measured to evaluate cell viability. All samples were done in triplicate (n=3), and data were reported as means with standard deviations. Absorbance measurements were carried out using a Synergy HT microplate reader (BioTek Instruments).

To determine the effect of radiation on cell viability, we plated cells in clear-bottom 96-well tissue culture–treated plates at a seeding density of approximately 10,000 cells/well in standard cell media. For each study group (in which radiation dose, treatment concentration, and time, respectively, were assessed), the cells were positioned in two 5×5 well squares per plate (i.e., wells including the area of A1:A5:E1:E5 and D8:D12:H8:H12) to diminish cross-irradiation and to achieve the best coverage for the 4 cm × 4 cm X-ray beam. The cells were allowed to adhere for 24 hours, and then half of the 25-well groups were treated with either 0.00 or 0.20 mM Au^{3+} and then incubated for 24 hours. Each 25-well group received a radiation dose of 0, 2, 4, 6, or 8 Gy. After X-ray irradiation, the parafilm was replaced with lids, and the cells were incubated for either 20 or 92 hours before an MTS assay was carried out as described above. Ten plates were used in this study, resulting in 25 wells per study group. Data were reported as means with standard errors.

JC-1 assay. JC-1 mitochondrial membrane potential stain was prepared according to the manufacturer's instructions. Cells were seeded in 8-well chambered coverglass slides and allowed 24 hours to adhere before Au^{3+} treatment with or without X-ray irradiation; control cells were not treated. Following treatment, the cells were washed 3 times with PBS, stained with JC-1 dye at a final concentration of 2 μM, and allowed to incubate for approximately 30 minutes. The cells were then washed three times with PBS, stained with Hoechst 33342 according to the manufacturer's instructions, and placed in fresh media in an incubator for approximately 30 minutes. The cells were then imaged using an SP8 Laser Scanning Confocal Microscope (Leica). A 488-nm excitation laser was used to excite the JC-1 stain; the emission filter settings described in the assay kit instructions and an avalanche photodiode photomultiplier tube detector were used. Cells were co-stained with Hoechst 33342 according to the manufacturer's protocol by incubation with Hoechst solution in PBS at 1:2,000 dilution for 30 minutes followed by imaging within 30 minutes. The 523- and 590-nm fluorescence per cell was quantified using Imaris software.

NADP/NADPH quantification assay. Standard and sample solutions for the NADP/NADPH quantification assay were prepared according to the manufacturer's instructions. Cells were seeded at equivalent densities in T-75 culture flasks for 24 hours before treatment with Au^{3+}. After treatment, the cells were lifted via trypsinization and seeded at equivalent densities in 35-mm culture dishes with full cell media. After 2 hours, the cells were irradiated with either 8 or 0 Gy. At 1 and 24 hours after irradiation, the cells were collected by trypsinization and washing in cold DPBS. Cell pellets ($\sim 4\times 10^6$ cells) were admixed with NADP/NADPH extraction buffer and incubated for 10 minutes. The cells were then separated from the supernatant containing NADP/NADPH by centrifugation at 10,000 *g* for 10 minutes. Each supernatant was separated

equivalently by volume to detect total NADP(H) (i.e., both $NADP^+$ and NADPH) as well as NADPH. For the detection of total NADP(H), the extracted solution was added directly to the 96-well plate. For the detection of NADPH only, $NADP^+$ was decomposed by heating the cell pellets at 60°C for 30 minutes and transferring them to a 96-well plate after any condensates were removed through centrifugation. The reaction mix, from the assay kit, was added to the sample and control wells, gently mixed, and incubated at room temperature for 5 minutes. Then, NADH developer was added to the wells and incubated at room temperature for 4 hours before absorbance measurements at 450 nm were collected with a Synergy HT microplate reader and quantified using a calibration curve (**Figure S12**).

Thiobarbituric acid reactive substance assay. Solutions for the thiobarbituric acid reactive substance (TBARS) assay were prepared according to the manufacturer's instructions. Cells were first seeded at equivalent densities in T-75 culture flasks and allowed 24 hours to adhere to the plate before Au^{3+} treatment; control cells were not treated. After treatment, the cells were lifted with trypsinization and seeded at equivalent densities in 35-mm culture dishes with full cell media. After 2 hours, the cells were irradiated with either 8 or 0 Gy. Twenty-four hours after irradiation, cell samples were lifted via trypsinization and collected for the TBARS assay. Lifted cells were washed with cold DPBS. Cell pellets ($\sim 1\times10^6$ cells) were admixed with lysis buffer and homogenized on ice using two 30-second rounds of tip sonication with a CPX 500 ultrasound probe sonicator (Cole-Parmer) at 25% amplitude. Following cell lysis, samples were centrifuged at 12,000 *g* for 2 minutes to remove cell debris. The supernatants were then admixed with thiobarbituric acid solution and incubated for 60 minutes at 95°C. After incubation, the samples were cooled in an ice bath for 10 minutes and placed in the wells of a 96-well plate along with

standards (**Figure S12**) for absorbance measurements at 532 nm using a Synergy HT microplate reader.

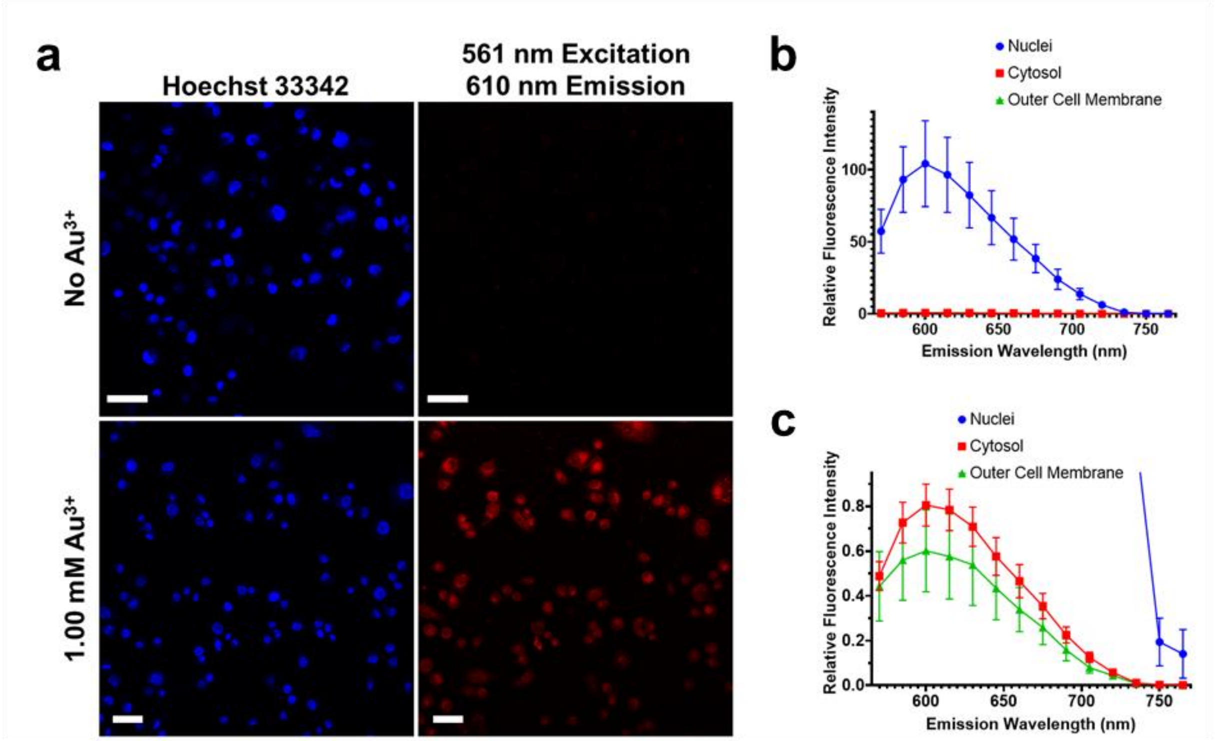

Figure S1. Confocal fluorescence imaging of GNC biomineralization in Au^{3+}-treated PANC1 cells compared to non-treated PANC1 cells and fluorescence emission spectra of GNCs. (a) Live PANC1 cells treated with 1.00 mM Au^{3+} for 24 hours and compared with untreated cells (i.e., no Au^{3+}). Cells were stained with Hoechst 33342 nuclear staining (left column, blue) ~30 minutes before imaging; the right column shows fluorescence from GNCs. (b and c) Emission fluorescence spectra of intracellular formed GNCs obtained from nuclei (blue), cytosol (red) and cytoplasmic cell membrane (blue). PANC1 cells were treated with 1.00 mM Au^{3+} for 24 hours and the emission spectra were collected from confocal fluorescence images obtained using 15 nm bandwidth cutoff emission filters. Fluorescence intensity values are shown as means with error bars indicating standard deviations (n=10 cells). Scale bars are 50 μm.

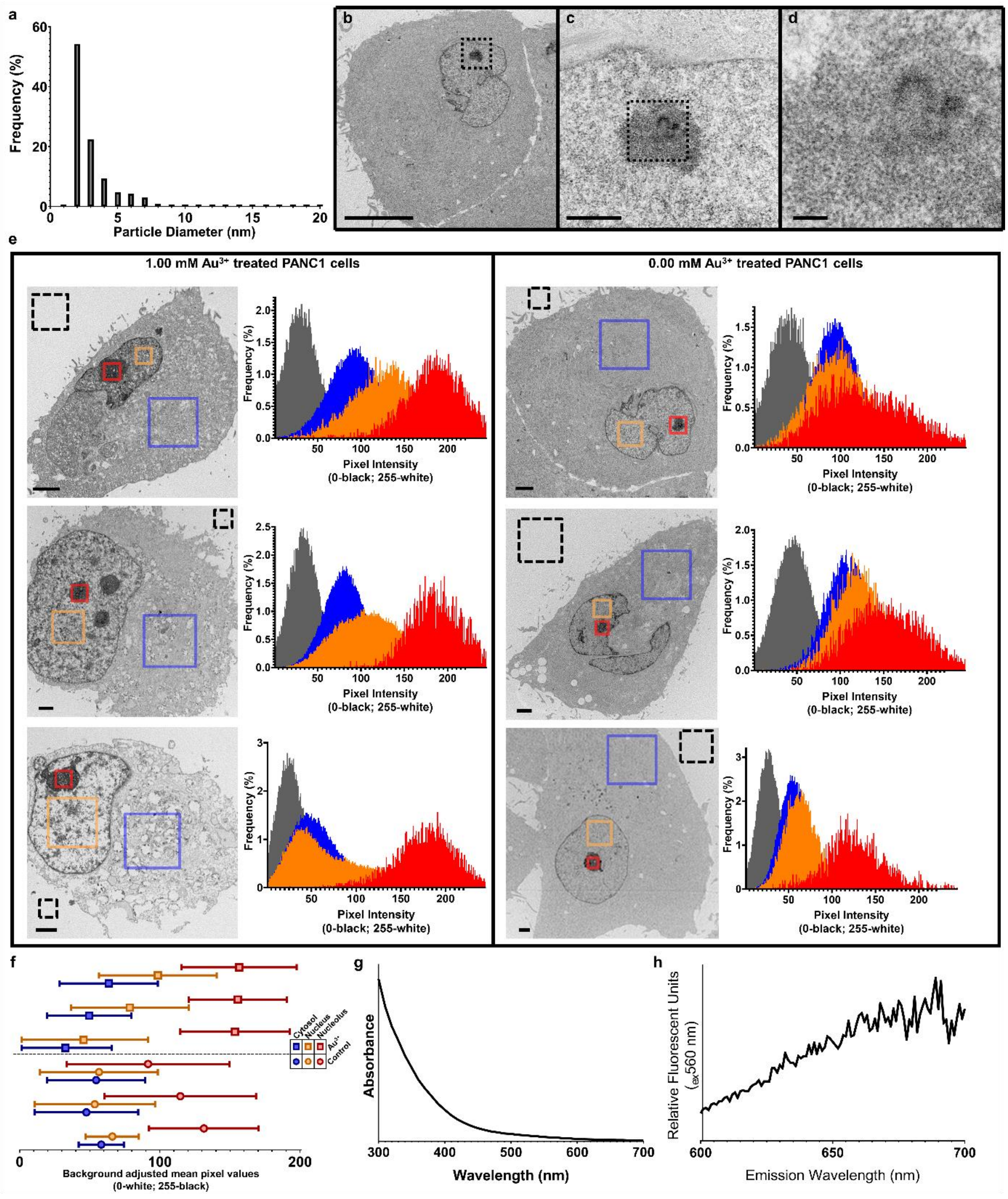

Figure S2. Analysis of cellular TEM and optical properties of albumin-coated GNCs. a, Histogram size distribution from ImageJ analysis of GNCs formed through the biomineralization of Au^{3+} by PANC1 cells and localized to the nucleolus. b-d, Transmission electron micrographs

of untreated PANC1 cells (negative control), with magnification of the nucleolus (d), for comparison with the chloroauric acid–treated cells in Fig. 1e-g. Scale bars in the left, middle, and right images are 10 μm, 1 μm, and 200 nm, respectively. e, Transmission electron micrographs of PANC1 cells with either 1.00 mM Au^{3+} or no Au^{3+} treatment with histogram pixel intensity frequencies for regions of interest (background, gray; cytoplasm, blue; nucleus, orange; and nucleolus, red) from ImageJ analysis. Scale bars are 10 μm. f, Mean pixel intensity values and standard deviations for regions of interest in the cell TEM images in (e). Optical properties of prefabricated albumin-coated GNCs prepared using the protocols described by Xie *et al.*[S6]: the absorbance spectrum (g) and fluorescence emission spectrum at 560 nm excitation (h).

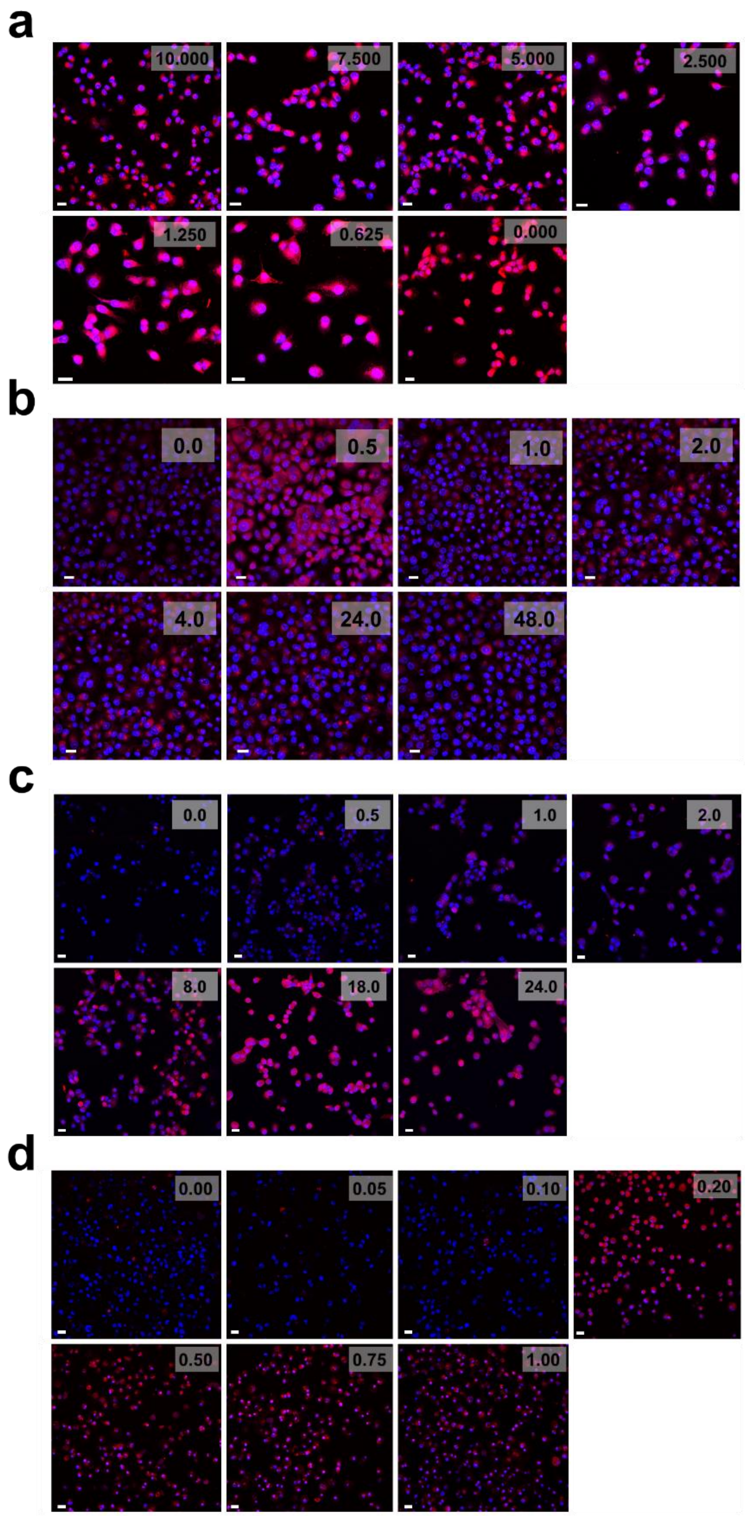

Figure S3. Representative confocal fluorescent images from optimization of the Au^{3+} treatment conditions. Live PANC1 cells that received 24-hour treatment with 1.0 mM

chloroauric acid with various concentrations of fetal bovine serum (inserts, %, v/v) in the incubating media (a); 24-hour treatment with 1.0 mM chloroauric acid in full cell media pre-conditioned with the cells for various times (insert, hours) (b); treatment with 1.0 mM chloroauric acid for various times (inserts, hours) in full cell media pre-conditioned with the cells for 24 hours (c); and 24-hour treatment with chloroauric acid at various concentrations (inserts, mM) in full cell media pre-conditioned with the cells for 24 hours (d). Cell nuclei were stained using Hoechst 33342 nuclear stain (blue). The fluorescence from GNCs was detected using 561 nm excitation and 610 nm emission. Scale bars are 25 μm.

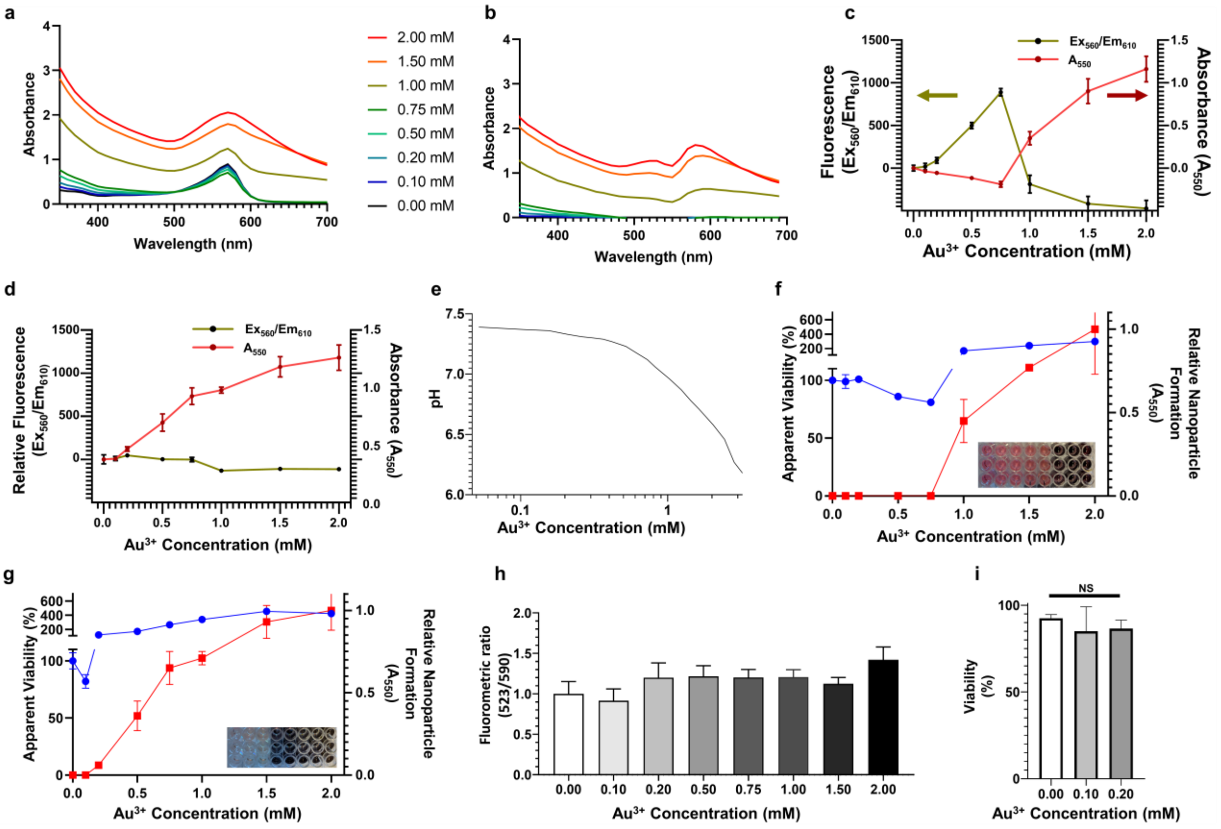

Figure S4. Cell viability. a and b, Raw (a) and corresponding background (*i.e.,* PANC1 cells in full growth media with 0.00 mM of Au^{3+}) subtracted absorbance spectra (b) of live PANC1 cells after 24-hour treatments with chloroauric acid at different concentrations in full cell growth media that was conditioned by the cells for 24 hours prior to Au^{3+} treatments. c and d, Quantification of ultrasmall fluorescent GNC and larger GNP formation as a function of the chloroauric acid concentration used to treat live PANC1 (c) and HPDE (e) cells in their corresponding full cell media under normal cell culture conditions. Data were obtained using microplate reader endpoint measurements of fluorescence with 560 nm excitation/610 nm emission (E_{610}) and absorbance at 550 nm (A_{550}) collected 24 hours after addition of chloroauric acid. Data points indicate means; error bars indicate standard deviations (n=3). e, Measured pH of full cell media (DMEM with 10% FBS) as a function of chloroauric acid concentration. f and g, Apparent viability (%; blue) of PANC1 cells (f) and HPDE cells (g) as measured by MTS assay and relative GNP formation (red)

as measured by absorbance at 550 nm prior to the addition of MTS solution as a function of Au^{3+} concentration. The inset photographs of the plates were captured immediately before the addition of MTS solution; the Au^{3+} concentration in the plates increases from left to right. h, Mean green/red fluorometric ratios of the JC-1 assay of PANC1 cells as a function of Au^{3+} concentration; the ratios were determined from confocal fluorescent images of labeled cells and were quantified using Imaris software. i, Viability (%) of PANC1 cells as a function of Au^{3+} concentration, quantified with AO/PI staining using Auto 2000 Cellometer cell counter (Nexcelom, MA, USA). Data points are mean values, and error bars are standard deviations (n=3);-$^{NS}P > 0.05$.

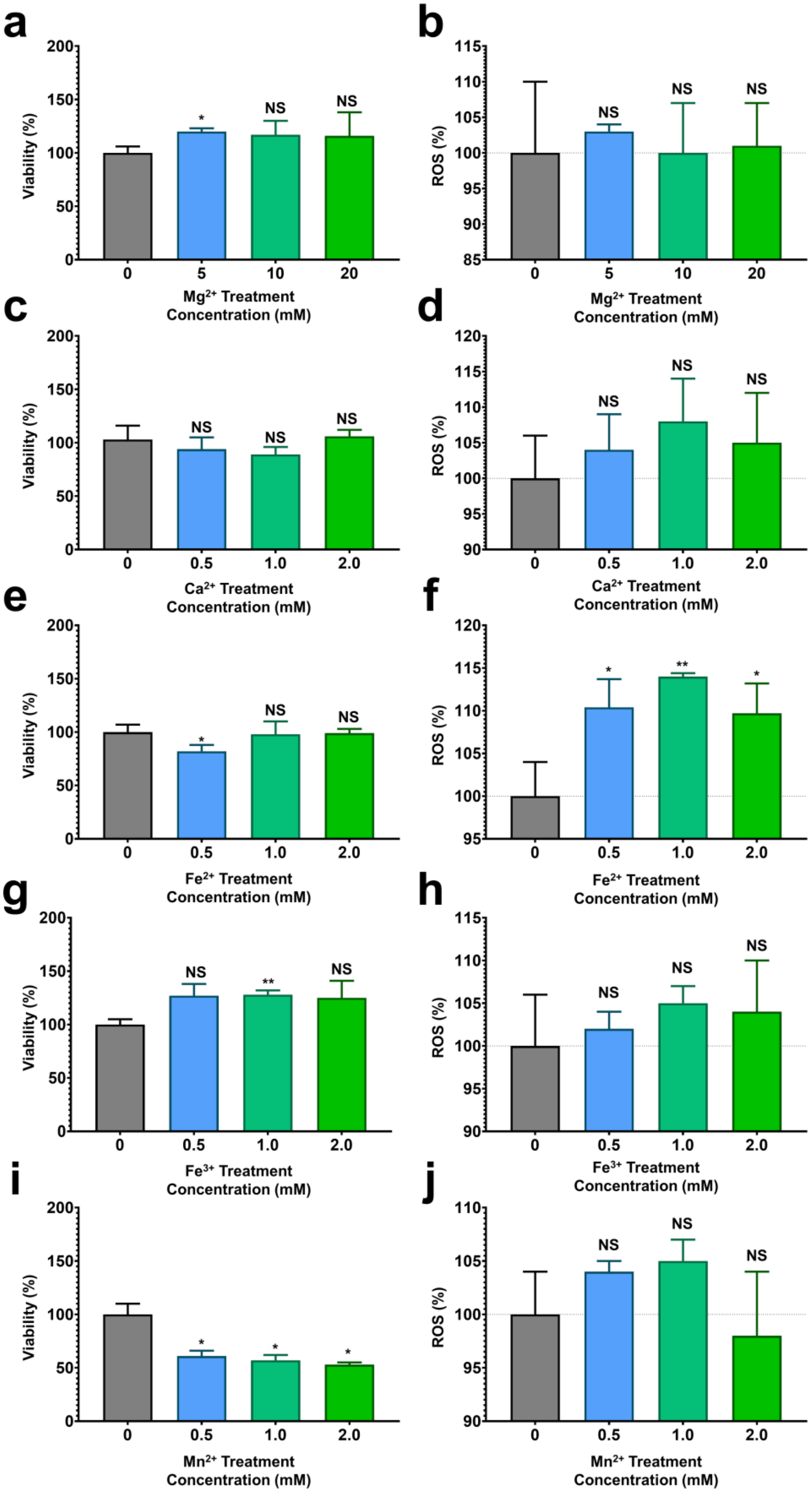
a
b
c
d
e
f
g
h
i
j
Viability (%)
ROS (%)
Mg²⁺ Treatment
Concentration (mM)
Ca²⁺ Treatment
Concentration (mM)
Fe²⁺ Treatment
Concentration (mM)
Fe³⁺ Treatment
Concentration (mM)
Mn²⁺ Treatment
Concentration (mM)
NS
*
**

Figure S5. Cell viability and ROS induction by physiological cationic ions. Viability (a, c, e, g, and i) and ROS formation (b, d, f, h, and j) in PANC1 cells after 24-hr treatments with Mg^{2+} (a and b), Ca^{2+} (c and d), Fe^{2+} (e and f), Fe^{3+} (g and h), and Mn^{2+} (i and j) at various physiologically relevant concentrations. Viability was assessed by MTS and ROS formation by Green assays. Data points are mean values, and error bars are standard deviations (n=3); $^{NS}P > 0.05$. $*P < 0.05$, $**P < 0.005$; by regular ANOVA.

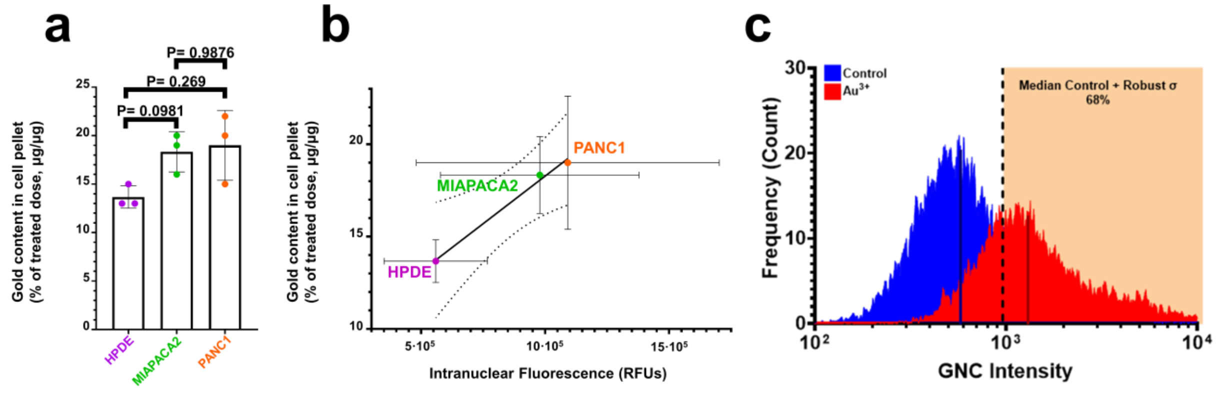

Figure S6. Uptake of gold ions. a, Mean gold content in pancreatic cells as a percent of the total added dose quantified by ICP-MS. Cells were treated with 0.20 mM Au^{3+} for 24 hours in full cell media (n=3). *P* values were calculated using Brown-Forsythe and Welch ANOVA tests for multiple comparisons. b, A plot of the correlation between mean intranuclear fluorescence (from Fig. 1h in the main text) and mean gold internalization (from the panel i) for pancreatic cells (n=3 confocal images). The line plot is a simple linear regression, and the dotted curved lines are the 90% confidence intervals (R^2=0.57; $P < 0.05$). c, Fluorescent intensity histograms of untreated (control – blue) and treated (0.20 mM Au^{3+}, 24 hours in full media – red) PANC1 cells from flow cytometry (n = 10,000 events). Median values are highlighted using darker solid lines and the GNC positive events are highlighted in orange to the right of the median of the control plus σ (marked with a dashed line).

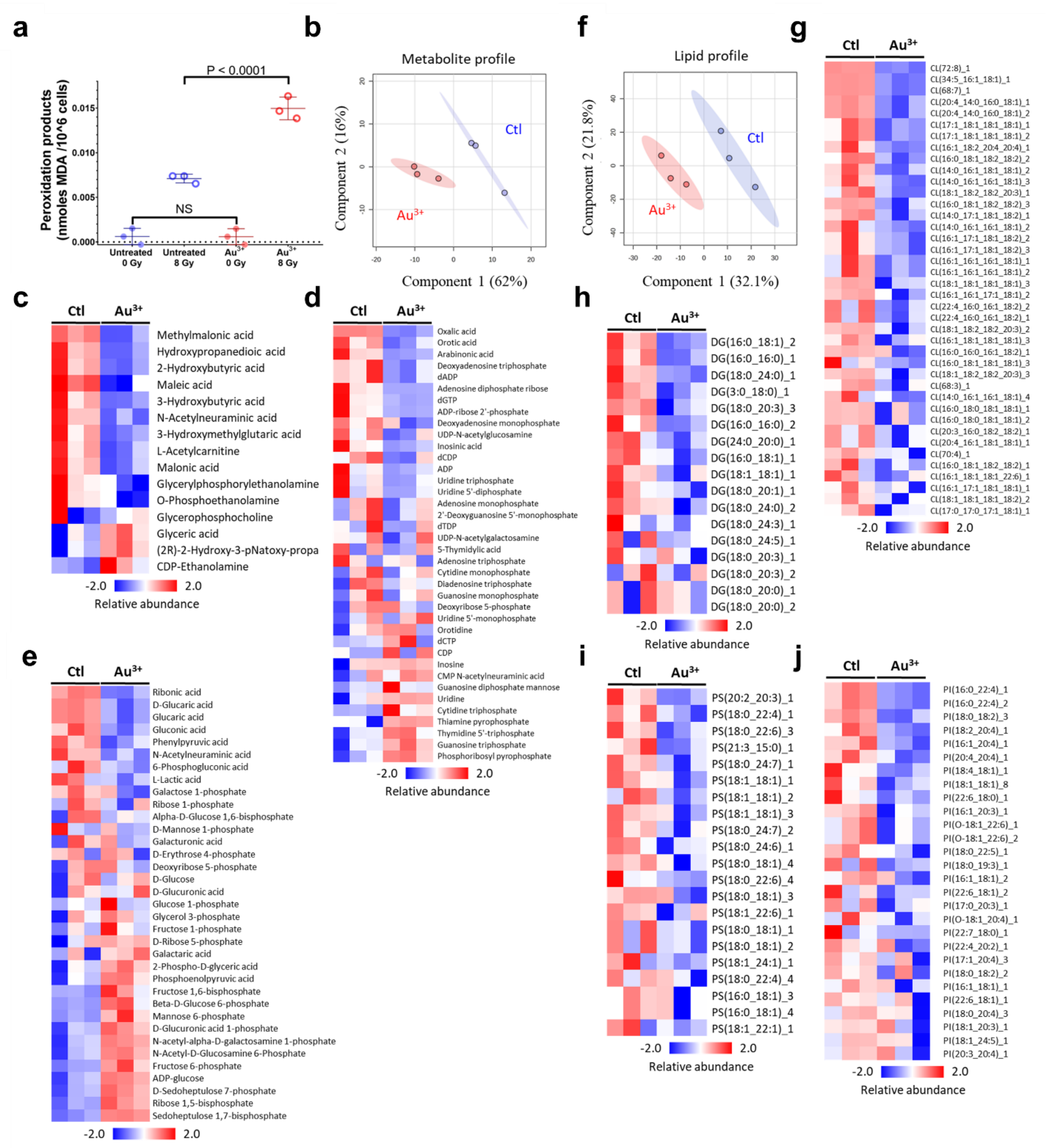

Figure S7. Formation of peroxidation products after RT of PANC1 cells treated with Au^{3+} ions and metabolic and lipidomic changes in PANC1 cells following Au^{3+} treatment. (a) Peroxidation product formation by PANC1 cells treated with combinations of Au^{3+} (0.00 or 0.20 mM) and radiation (0 or 8 Gy) 24 hours after irradiation as assessed with a TBARS assay. Cells

were allowed 24 hours to condition the media prior to treatment. Au^{3+} treatments were applied over 24 hours in full cell media with normal incubation parameters. Data points are mean values, and error bars are standard deviations (n=3, $^{NS}P > 0.05$). (b) Global metabolite profile differences in PANC1 cells with or without 0.2 mM Au^{3+} treatment by principal component analysis. (c-e) Heatmaps showing the relative abundance of metabolites in PANC1 cells with or without 0.20 mM Au^{3+} treatment that are involved in metabolic networks associated with short chain fatty acids (c), DNA damage (d) and glycolysis and pentose phosphate pathways (e). (f) Global lipid profile differences in PANC1 cells with or without 0.2 mM Au^{3+} treatment by principal component analysis. (g) Heatmap showing the relative abundance of metabolites involved in mitochondrial oxidation called cardiolipin networks in PANC1 cells with or without 0.20 mM Au^{3+} treatment. (h-j) Heatmaps showing the relative abundance of metabolites involved in lipid-mediated signaling by diacylglycerol (DG) (h), phosphatidylserine (PS) (i), and phosphatidylinositol (PI) (j) networks in PANC1 cells with or without 0.20 mM Au^{3+} treatment.

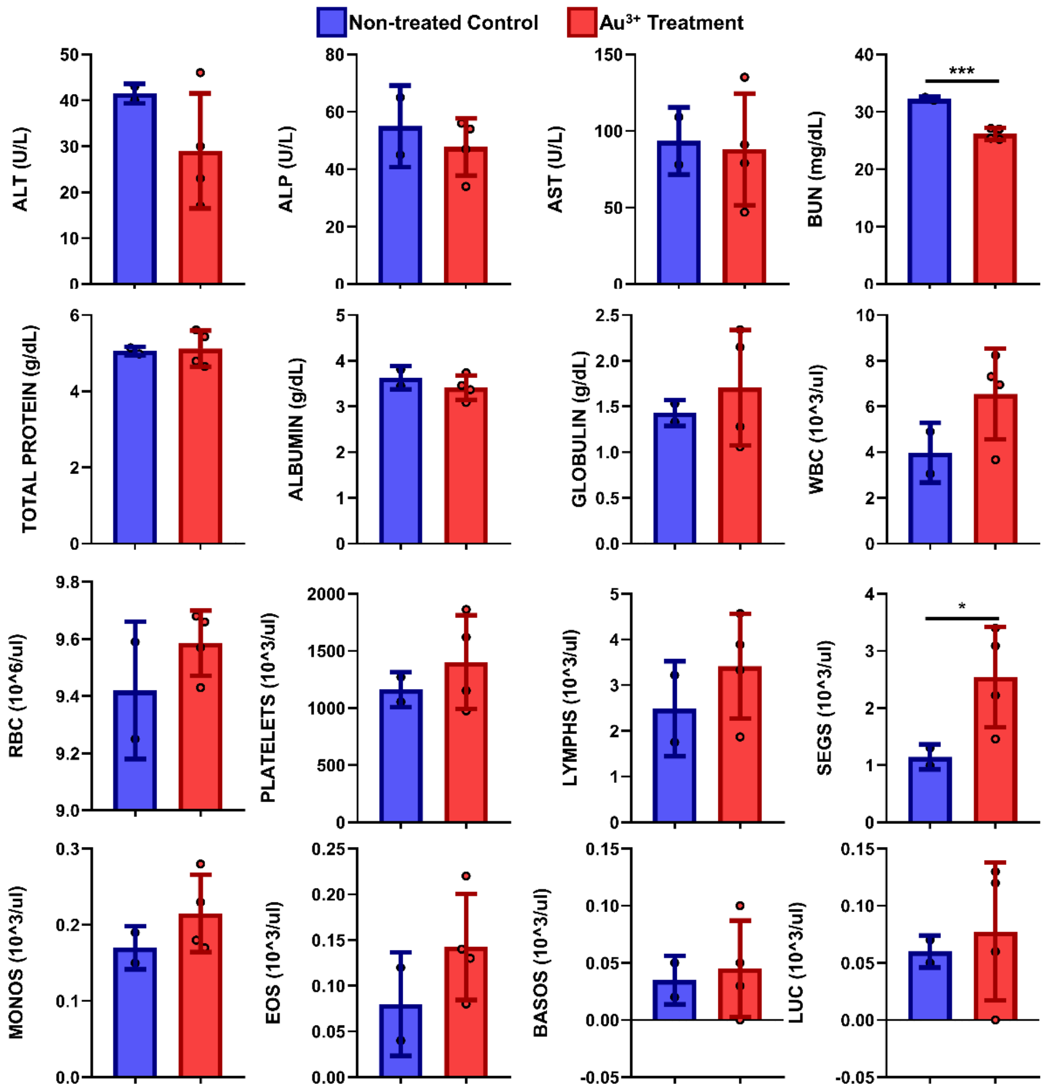

Figure S8. Biochemistry and hematology panels following intratumoral treatments of 0.00 mM and 1.00 mM Au^{3+}. Toxicity assessment. Chemistry and hematology panels comparing the levels of alanine transaminase (ALT), alkaline phosphatase (ALP), aspartate transaminase (AST), blood urea nitrogen (BUN), total protein, albumin, globulin, and counts of white blood cells (WBC), red blood cells (RBC), platelets, lymphocytes (LYMPHS), neutrophils (SEGS), monocytes (MONOS), eosinophils (EOS), basophils (BASOS), and large undifferentiated cells (LUC) in blood collected from nu/nu mice with hind flank PANC1 tumor xenografts 48 hours after

treatment with either 0.00 or 1.0 mM Au^{3+}. *$P < 0.05$, ***$P < 0.001$; unpaired t-test with Welch's correction.

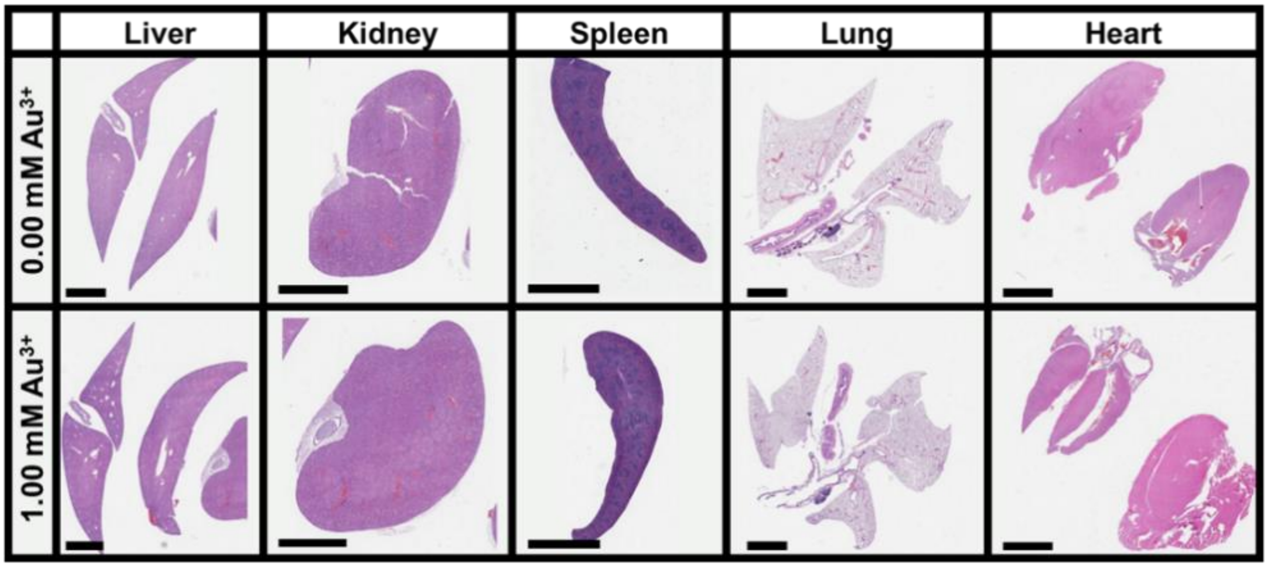

Figure S9. Histology of normal organs after intratumoral treatments of 0.00 mM and 1.00 mM Au^{3+}. Representative histology images of normal organs (liver, kidney, spleen, lung, and heart) from *nu/nu* mice with PANC1 xenografts that received intratumoral injections of either 0.00 or 1.00 mM Au^{3+} 48 hours before euthanasia. Scale bars are 3 mm.

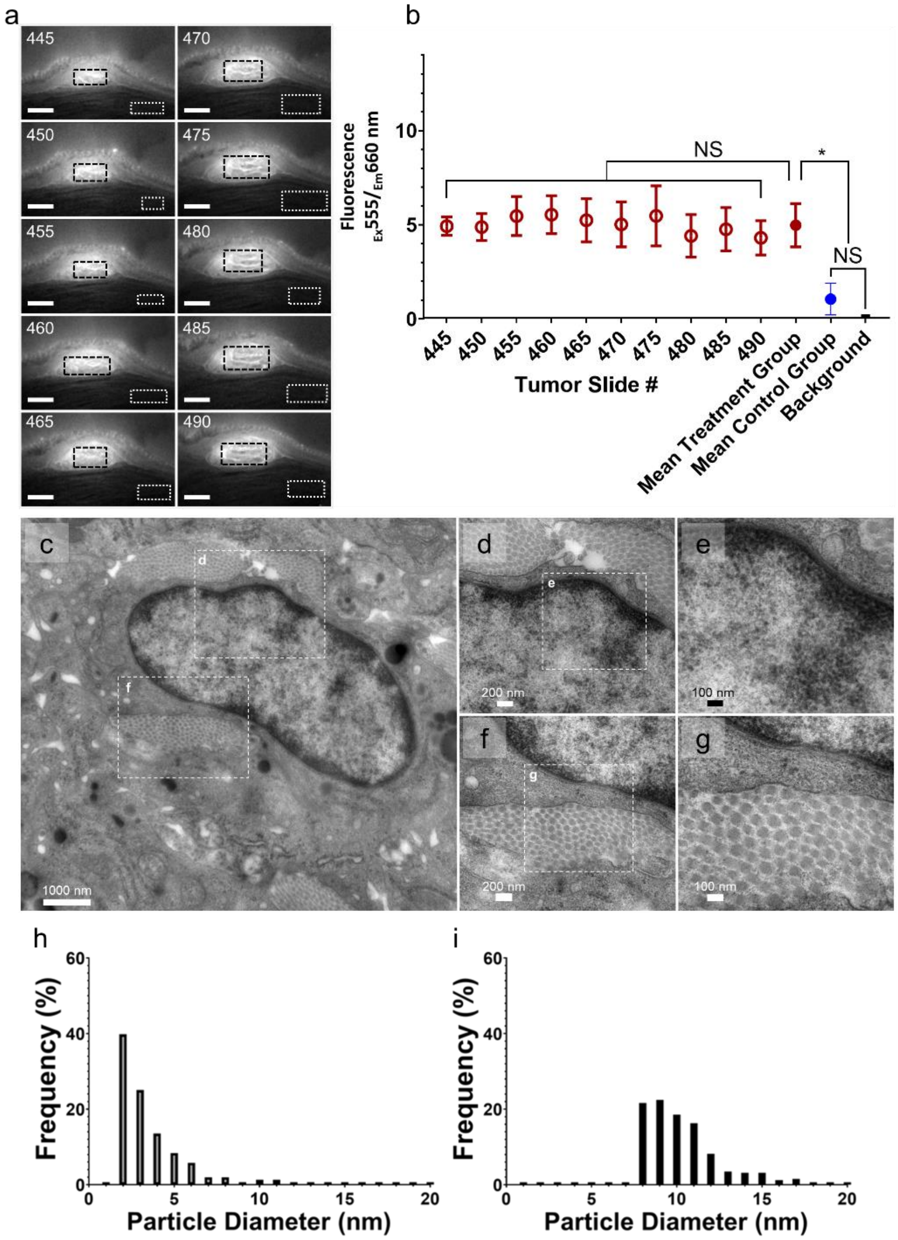

Figure S10. Intratumoral GNC biomineralization. a and b, Cryo-fluorescence images of single tumor slices (a) with the mean tumor fluorescence value (± standard deviation) per slice compared with the mean tumor fluorescence value of all slices and the background tissue autofluorescence (b). The tumor fluorescence values for individual slices were not significantly distinct (NS, $P >$ 0.5) from each other or from the mean tumor fluorescence value for all slices, and all mean tumor

fluorescence values were significantly distinct from the background tissue fluorescence values (*, $P < 0.5$; multiple unpaired t-tests). c-g, TEM of a PANC1 xenograft tumor harvested from a nu/nu mouse 48 hours after intratumoral injection of 0.00 mM Au^{3+} in PBS (negative control) (c), with magnified views of the nucleolus (d and e) and collagen fibers (f and g). g and i, Histogram size distribution from ImageJ analysis of gold nanoparticles formed through the biomineralization of Au^{3+} inside PANC1 tumor xenografts and localized to the nucleolus (g) or collagen (i).

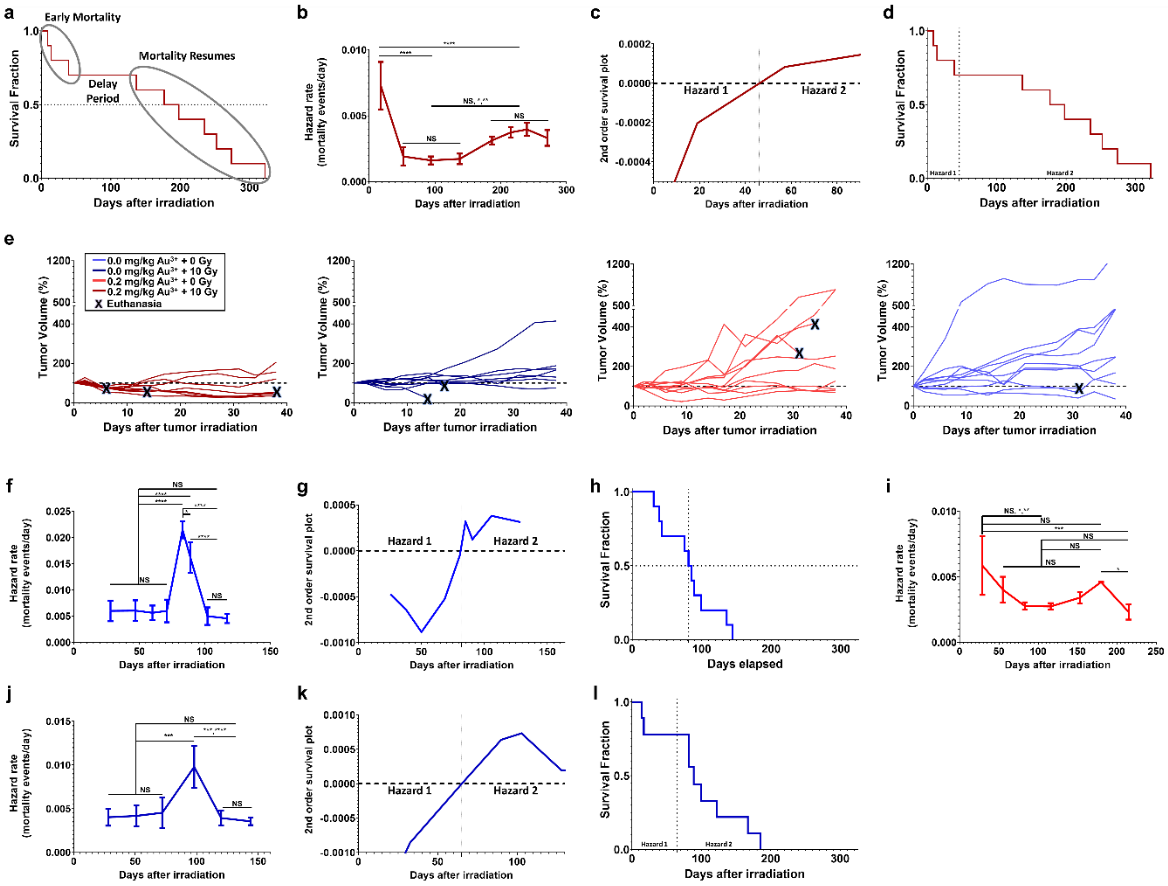

Figure S11. Analysis of competitive risk events. a-d, Combined Au^{3+} and radiation treatment group: initial Kaplan-Meyer survival analysis with regions of interest circled (a); mortality rate across time (b); second derivative inflection point test (Y=0 intercept highlighted at ~46 days) (c); and the inflection time point between the two hazards highlighted on the initial survival plot (d). e, Normalized tumor volume measurements over time for individual mice receiving different treatments with Au^{3+} (0.0 or 0.2 mg/kg) and radiation (0 or 10 Gy); each mortality event within the first 38 days of observation is marked with an “X”. f-h, Non-treatment control group: mortality rate across time (f); second derivative inflection point test (Y=0 intercept highlighted at 82 days) (g); and the inflection time point and the median survival highlighted on the initial survival plot (h). i, Mortality rate of the Au^{3+}-only treatment group across time. j-l, Radiation-only treatment

group: mortality rate across time (f); second derivative inflection point test (Y=0 intercept highlighted at 65 days) (g); and the inflection time point highlighted on the initial survival plot (h). The plots of mortality rate across time (b, f, i, and j) were calculated by four-point averaging. Error bars are standard deviations. $^{NS}P > 0.05$, $*P < 0.05$, $**P < 0.01$, $***P < 0.001$, and $****\ P < 0.0001$.

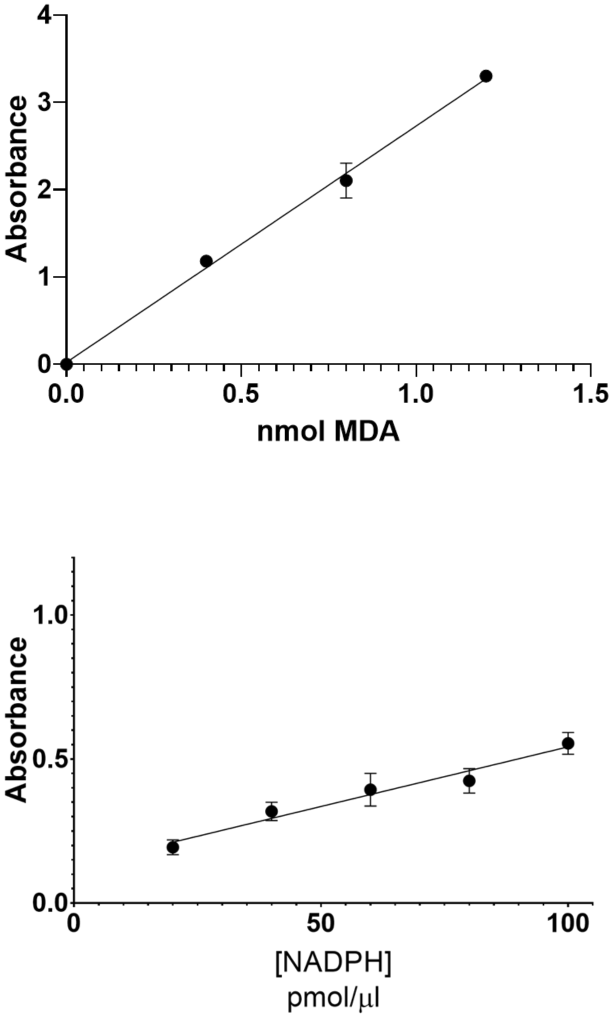

Figure S12. Assay calibration plots. Calibration of the TBARS assay (top) and NADP(H) assay (bottom). Error bars are standard deviations.

Table S1. Histopathology evaluation results

**Treatment Group**	**Sham Dose**	**Sham Dose**	**Summary**	**High Dose**	**High Dose**	**High Dose**	**High Dose**	**Summary**
**Organ Morphologic Diagnosis**	**Lesion Grade**	**Lesion Grade**	**Incidence of lesions**	**Lesion Grade**	**Lesion Grade**	**Lesion Grade**	**Lesion Grade**	**Incidence of lesions**
Liver	N	N	2	N	N	A	A	4
Extramedullary hematopoiesis, perivascular and portal areas			0			2	1	2
Microgranulomas, focal			0					0
Gall Bladder	N	N	2	A	N	N	N	4
Inflammation, histiocytic, subacute			0	1				1
Kidneys	N	N	2	A	N	N	A	4
Lymphohistiocytic infiltration, perivascular and interstitial			0	1			2	2
Hypertrophy, focal, epithelium, proximal renal tubules			0					0
Proteinaceous cast, tubules lumen (incidental finding)			0					0
Lung	A	N	2	A	N	A	A	4
Increased number of alveolar macrophages, diffuse	1		1			1		1

Lymphohistiocytic infiltration, perivascular and peribronchiolar			0			3	1	2
Trachea	N	N	2	N	N	A	N	4
Inflammation, trachea (tracheitis)			0			1		1
Esophagus	N	N	2	N	N	N	N	4
			0					0
Heart	N	N	2	N	N	N	N	4
			0					0
Skeletal muscle	N	N	2	N	N	N	A	4
Inflammation, focal			0				1	1
Aorta	N	N	2	0	N	N	N	4
			0					0
Spleen	A	A	2	A	A	A	A	4
Lymphocytic hyperplasia	2	2	2	4	1	3	1	4
Extramedullary hematopoiesis, increased			0	3	1	2		3
Plasmacytosis			0	2		2	2	3
Mesenteric lymph nodes and mesenterium	A	A	2	A	A	A	A	4
Lymphocytic hyperplasia	2	4	2	2	1	3	2	4
Plasmacytosis		2	1	1	1	4	1	4
Histiocytosis	3	4	2	2	2	3	3	4
Inflammation, pyogranulomatous with multinucleated giant cells and atypical cells, adipose tissue, mesentery			0	4				1
Thymus	N	0	1	N	N	N	N	4

Rudimentary thymus with epithelial cysts formation (normal for nu/nu mice)	P			P	P	P	P	
Tumor	P	P	2	P	P	P	P	4
Inflammation, lymphohistiocytic and less neutrophilic, peripheral	3	4	2	4	4	4	4	4
Intravascular tumor metastasis				P				
Necrosis of tumor, central (percent)	25	25	25	20	5	15	45	21

N, normal tissue; A, abnormal tissue; P, present but unscored lesion; 0, number of events.

Lesions were graded on a scale of 1 to 4, with grade 1 indicating a minimal, infrequent, or barely noticeable tissue change (1-10% of tissue affected); grade 2 indicating a mild, slight, sporadic, noticeable but not prominent tissue lesion (11-20% of tissue affected); grade 3 indicating a moderate, frequent, typical, common, prominent tissue lesion (21-40% of tissue affected); and grade 4 indicating a marked, extensive, numerous, severe, overwhelming tissue lesion (41-100% of tissue affected).

AUTHOR INFORMATION

Corresponding Author

*To whom correspondence should be addressed: E-mail: krishnan.sunil@uth.tmc.edu and ksokolov@mdanderson.org

Author Contributions

The manuscript was written through contributions of all authors. Conceptualization of the work was by ASD, SK, and KVS; methodology design by ASD, YM, IM, PL, MG, SK, and KVS; investigation carried out by ASD, YM, IM, PL, and MG; visualization of data by ASD, IM, PL, and MG; supervision by SK and KVS; writing for the original draft carried out by ASD, YM, IM, PL, MG, SK, and KVS; and the review & editing done by ASD, YM, IM, PL, MG, SK, and KVS. All authors have given approval to the final version of the manuscript.

Funding Sources

This work was supported by the National Institutes of Health (NIH) through grants to K.V.S. (R21CA252156 and R01CA274415) and S.K. (R21CA252156, R01CA257241, and R01DE028105). A.S.S.-D. was supported by a fellowship funded by an NIH Institutional National Research Service Award (T32 CA196561).

ACKNOWLEDGMENT

This work used MD Anderson's High-Resolution Electron Microscopy Facility (supported in part by NIH P30CA016672), Advanced Microscopy Core (NIH S10RR029552), Department of Veterinary Medicine and Surgery Veterinary Pathology Services, Cytogenetics and Cell Authentication Core (NIH P30CA016672), Small Animal Imaging Facility (NIH P30CA016672), Flow Cytometry and Cellular Imaging Core Facility (P30CA16672), and Metabolomics Facility (supported in part by Cancer Prevention Research Institute of Texas [CPRIT] grant number RP130397 and NIH grants S10OD012304-01, U01CA235510, and P30CA016672). We thank Joe Munch in MD Anderson's Research Medical Library for editing the manuscript, and Dmitry Nevozhay, Department of Imaging Physics, M.D. Anderson Cancer Center, for fruitful discussions of statistical analysis of survival studies.

ABBREVIATIONS

GNP, gold nanoparticle; GNC, gold nanocluster; Au^{3+}, ionic gold from chloroauric acid; TEM, transmission electron microscopy; FBS, fetal bovine serum; ICP-MS, inductively coupled plasma mass spectrometry; JC-1, tetraethylbenzimidazolylcarbocyanine iodide; NADP, Nicotinamide adenine dinucleotide phosphate; TBARS, thiobarbituric acid reactive substance; CT, computed tomography; PBS, phosphate-buffered saline; H&E, hematoxylin and eosin; UHPLC-HRMS, ultra-high resolution mass spectrometry.

REFERENCES

S1. Liu, H.; Liu, Z.; Wang, Y.; Xiao, J.; Liu, X.; Jiang, H.; Wang, X., Intracellular Liquid-Liquid Phase Separation Induces Tunable Anisotropic Nanocrystal Growth for Multidimensional Analysis. *Advanced Functional Materials* **2023**, 2302136.
S2. Austin, P. C.; Lee, D. S.; Fine, J. P., Introduction to the analysis of survival data in the presence of competing risks. *Circulation* **2016,** *133* (6), 601-609.
S3. Satagopan, J.; Ben-Porat, L.; Berwick, M.; Robson, M.; Kutler, D.; Auerbach, A., A note on competing risks in survival data analysis. *British journal of cancer* **2004,** *91* (7), 1229-1235.
S4. Prinja, S.; Gupta, N.; Verma, R., Censoring in clinical trials: review of survival analysis techniques. *Indian journal of community medicine: official publication of Indian Association of Preventive & Social Medicine* **2010,** *35* (2), 217.
S5. Sly, L.; Blackall, L. L.; Kraat, P.; Tian-Shen, T.; Sangkhobol, V., The use of second derivative plots for the determination of mol% guanine plus cytosine of DNA by the thermal denaturation method. *Journal of microbiological methods* **1986,** *5* (3-4), 139-156.
S6. Xie, J.; Zheng, Y.; Ying, J. Y., Protein-directed synthesis of highly fluorescent gold nanoclusters. *Journal of the American Chemical Society* **2009,** *131* (3), 888-889.
